# Contribution of syndecans to cellular internalization and fibrillation of amyloid-β(1–42)

**DOI:** 10.1038/s41598-018-37476-9

**Published:** 2019-02-04

**Authors:** Tamás Letoha, Anett Hudák, Erzsébet Kusz, Aladár Pettkó-Szandtner, Ildikó Domonkos, Katalin Jósvay, Martin Hofmann-Apitius, László Szilák

**Affiliations:** 1 Pharmacoidea Ltd., Szeged, H-6726 Hungary; 2 Biological Research Centre of the Hungarian Academy of Sciences, Szeged, H-6726 Hungary; 3 Fraunhofer Institute for Algorithms and Scientific Computing (SCAI), Sankt Augustin, 53754 Germany; 4 Szilak Laboratories, Bioinformatics and Molecule-Design, Szeged, H-6723 Hungary

## Abstract

Intraneuronal accumulation of amyloid-β(1–42) (Aβ1–42) is one of the earliest signs of Alzheimer’s disease (AD). Cell surface heparan sulfate proteoglycans (HSPGs) have profound influence on the cellular uptake of Aβ1–42 by mediating its attachment and subsequent internalization into the cells. Colocalization of amyloid plaques with members of the syndecan family of HSPGs, along with the increased expression of syndecan-3 and -4 have already been reported in postmortem AD brains. Considering the growing evidence on the involvement of syndecans in the pathogenesis of AD, we analyzed the contribution of syndecans to cellular uptake and fibrillation of Aβ1–42. Among syndecans, the neuron specific syndecan-3 isoform increased cellular uptake of Aβ1–42 the most. Kinetics of Aβ1–42 uptake also proved to be fairly different among SDC family members: syndecan-3 increased Aβ1–42 uptake from the earliest time points, while other syndecans facilitated Aβ1–42 internalization at a slower pace. Internalized Aβ1–42 colocalized with syndecans and flotillins, highlighting the role of lipid-rafts in syndecan-mediated uptake. Syndecan-3 and 4 also triggered fibrillation of Aβ1–42, further emphasizing the pathophysiological relevance of syndecans in plaque formation. Overall our data highlight syndecans, especially the neuron-specific syndecan-3 isoform, as important players in amyloid pathology and show that syndecans, regardless of cell type, facilitate key molecular events in neurodegeneration.

## Introduction

Dementia is one of the most important health-care problems in ageing populations^1,2^. The most frequent cause of dementia is Alzheimer’s disease (AD)^3,4^. Induced by widespread neurodegeneration, AD is a rapidly growing epidemic affecting about 30 million individuals worldwide^5^. Beginning with insidious deterioration of higher cognition that progresses to severe dementia, currently untreatable AD represents a major unmet medical need^6,7^.

There is compelling evidence that amyloid beta peptide (Aβ), rendered in β-sheet dominated senile plaques, is a hallmark of AD^8^. Although the precise role of Aβ in AD is not fully understood, plaque formation of accumulated Aβ is still considered to be a central event in disease development^9,10^. The accumulation of Aβ arises from the imbalance between its production and clearance^11^. Recent evidence in humans suggest that faulty Aβ clearance has a profound influence in the pathogenesis of AD^12^. Cellular studies shows that neuronal endocytic and lysosomal systems become damaged early in AD, leading to the intraneuronal build-up of Aβ that precedes extracellular plaque formation^13–19^. Intracellular accumulations of the aggregation-prone Aβ1–42 isoform then promotes cell-to-cell transfer, also an early cellular event that is seemingly independent of later appearances of cellular toxicity^20^. Thus, cellular uptake of Aβ1–42 has profound influence on the course of AD.

Interaction of heparan sulfate proteoglycans (HSPGs) with Aβ has been well documented^21–25^. HSPGs have been implicated in several pathogenic features of AD, including its colocalization with amyloid plaques^26–29^. Binding of Aβ1–42 to HSPGs is mediated by electrostatic interactions arising between the negatively charged heparan sulfate (HS) chains and the cationic heparin-binding motif of Aβ1–42^30,31^. Attachment of Aβ1–42 to HS chains induces its multimerization, leading to the formation of toxic fibrillar aggregates^32–34^. Fibrillation of Aβ1–42 meanwhile increases its interactions with HSPGs, such as syndecan-4 (SDC4), the universally expressed isoform of the syndecan (SDC) family of transmembrane proteoglycans^35^. Besides SDC4, number of other HSPGs, including SDC3, the neuron specific isoform of the SDC family, are significantly increased in human AD brains and through modulating Aβ aggregation and clearance they promote amyloid pathology^11^. In other studies, SDC1-3 were also found to be associated with the majority of senile plaques in AD brains^36^.

Due to their highly sulfated polyanionic glycosaminoglycan (GAG) chains, SDCs interact with myriad of extracellular cationic ligands and transmit signals from the extracellular space towards the cellular interior, hence influencing cellular metabolism, transport and information transfer^37–39^. As key regulators of cell signaling and biological functions, SDCs also play important roles in the pathogenesis of several human diseases^40–42^. Expression of SDCs follow a tissue specific pattern: SDC1 is widely expressed in epithelial and plasma cells, SDC2 in mesenchymal cells, whereas SDC3 is mainly localized to neural tissues. In contrast to other SDCs, SDC4 is abundant in many cell types^38,43^. SDCs share similar structure: a conserved short, one span transmembrane domain (TM) and the approximately 30 amino acid length cytoplasmic domain (CD). Through their CDs, SDCs effect a large number of signaling cascades^44^. The N-terminal extracellular domains (ectodomains) of SDCs contain three GAG attachment sites for HS near the N terminus and may bear chondroitin sulfate (CS) at the juxtamembrane region^45^. The SDC4 ectodomain also compromises a cell-binding domain (CBD) mediating cell to cell attachment^46^. SDCs also mediate cellular uptake of a wide variety of macromolecules, along with viruses and bacteria^47–50^. During endocytosis, ligand- or antibody-mediated clustering induces the redistribution of SDCs to lipid rafts and stimulation of a lipid raft-dependent, but clathrin- and caveolae-independent endocytosis of the SDC-ligand complex^51^. Contrary to SDCs, the cell surface HSPG glypicans mediate internalization of their ligands primarily through caveolin-dependent endocytosis^52^.

Due to their involvement in endocytosis, SDCs are attractive targets to deliver macromolecules into the cells^53–55^. Throughout the years, our research group has been exploring the SDC-mediated uptake of cell-penetrating peptides (CPPs), small cationic peptides enabling the efficient intracellular delivery of membrane-impermeable biomolecules^56–59^. Cationic CPPs such as Tat, penetratin or polyarginine all utilize SDC-dependent macropinocytic pathways to enter the cells. Since CPPs internalized via SDC-mediated routes can bypass lysosomal degradation, aiding CPP-attached cargoes to exert their bioactivity intracellularly, SDC-mediated uptake and intracellular accumulation of fibrillar Aβ1–42 aggregates could have indeed damaging effects on neurons. The different expression pattern of SDCs in the CNS also makes SDCs exciting molecular targets to be explored in the course of neurodegeneration^60^.

Considering the evidence on the potential involvement of SDCs in membrane attachment and subsequent cellular delivery of Aβ1–42, we decided to analyze the internalization of Aβ1–42 in cellular models expressing distinct SDC isoforms. In order to analyze SDCs’ effect of without the interference of other HSPGs or caveolae-mediated endocytosis, we chose the K562 cell line – a human myelogenous leukemia cell line with reportedly no expression of any members of the syndecan or glypican HSPG families, along with no caveolae expression – as model cells to express the various SDC isoforms^61–64^. Quantitative uptake assays with SDC transfectants enabled quantitative assessment of SDCs’ contribution to Aβ1–42 uptake, while studies with SDC structural mutants revealed the role of SDC domains in interaction with the peptide. Utilization of Thioflavin T (ThT) fluorescence assays and scanning electron microscopy also helped to assess the effect of SDCs on Aβ1–42 fibrillation, the molecular mechanism responsible for the formation of toxic amyloid oligomers. Observations obtained on stable SDC transfectants (established in K562 cells) were also confirmed in SH-SY5Y neuronal cells.

Overall our data highlight SDCs, especially the neuron-specific SDC3 isoform, as important players in the amyloid pathology of AD and show that SDCs, regardless of cell type, facilitate key molecular events in neurodegeneration.

## Results

### Contribution of SDCs to Aβ1–42 uptake and fibrillation

**Figure 1:**
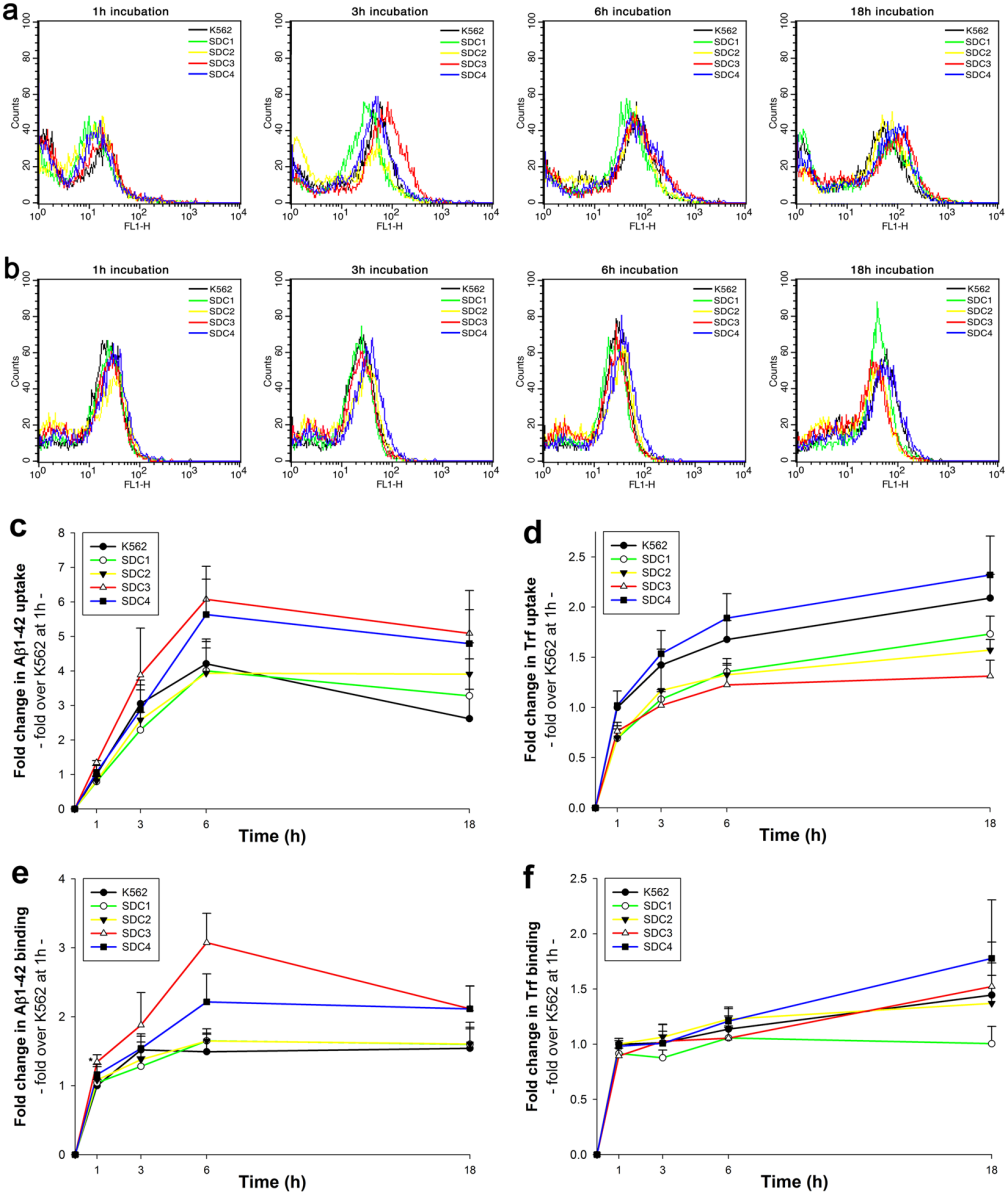
Kinetics of Aβ1–42 and Trf uptake. WT K562 cells and SDC transfectants were incubated with FITC-labeled Aβ1–42 or Trf for 1, 3, 6 and 18 h at 37 °C. Cellular uptake and attachment was analyzed with flow cytometry. (**a**,**b**) Flow cytometry histograms showing kinetics of intracellular fluorescence following incubation with fluorescent Aβ1–42 and Trf, respectively. (**c**–**f**) Detected intra- and extracellular fluorescence intensities of Aβ1–42 or Trf-treated cells were normalized to those of WT K562 cells treated with the respective proteins (either Aβ1–42 or Trf) for 1 h. The lines represent mean ± SEM of four independent experiments.

While cell surface HSPGs have been already identified as key targets for the cellular attachment and internalization of Aβ1–42, the exact contribution of transmembrane SDCs to Aβ1–42 endocytosis has not been assessed yet. To enable the exact assessment of SDCs’ contribution to cellular uptake of Aβ1–42, while minimizing the interfering effects of other HSPGs or caveolae-mediated endocytosis, stable transfectants of SDCs were created in the K562 cells, a cell line with reportedly low HSPG and no syndecan or glypican expression, along with no detectable levels of caveolin-1, the main component of caveolae^61–65^. The low expression of membrane HSPGs (see Supplementary Fig. S1. for comparison of HS expression of K562 cells to that of SH-SY5Y, a widely used neuronal cell line) and the inability to form caveolae, the source of caveolar endocytosis, makes K562 an ideal human model cell line to study the effect of SDC overexpression on Aβ1–42 uptake without the interference of other HSPGs or caveolae-mediated endocytosis. As the role of HS in the cellular attachment of Aβ1–42 has been already demonstrated, SDC transfectants were standardized according to their HS expression (Supplementary Fig. S2). (It is worth noting that contrary to HS, we could not detect any CS expression on wild-type K562 cells and SDC transfectants [Supplementary Fig. S3]). Thus SDC transfectants with similar level of HS expression were selected and, along with wild-type (WT) K562 cells, treated with FITC-labeled Aβ1–42 or transferrin (Trf), the marker of clathrin-mediated endocytosis, for 1, 3, 6 and 18 hours. Contribution of SDCs to cellular uptake and binding of FITC-Aβ1–42 or FITC-Trf was then quantified with quantitative flow cytofluorometric assays. Protein internalization was assessed by adding trypan blue (dissolved at a concentration of 0.25% in ice-cold 0.1 M citrate buffer) 1 min before the analyses, thus extracellular fluorescence of surface bound fluorescent proteins (Aβ1–42 or Trf) was quenched^66^. Extracellularly attached proteins were quantified by the difference of the fluorescence measures of protein-treated samples unquenched and quenched with trypan blue. As Fig. 1. shows, internalization of Aβ1–42 was highest in SDC3 transfectants at every time points. SDC4 transfectants surpassed WT K562 cells in uptake of Aβ1–42 after 6 h of incubation, while SDC2 bested K562 cells in uptake and attachment of Aβ1–42 only at 18 h. Compared to K562 cells, cellular uptake of Aβ1–42 into SDC1 transfectants were lower until 18 h of incubation. On the other hand, uptake of Trf, the marker of clathrin-mediated endocytosis, was higher in K562 cells than in SDC1, 2 and 3 transfectants, while SDC4 exhibited slightly higher Trf uptake levels than WT K562 cells. Attachment of Aβ1–42 was highest in SDC3 transfectants and peaked at 6 h of incubation. Extracellular attachment of Aβ1–42 in SDC4 transfectants was also higher compared to WT K562 cells, while both SDC1 and SDC2 exhibited similar extracellularly attached Aβ1–42 levels as WT K562 cells. In case of Trf, attachment of all cells were quite similar, except for SDC1 that seemed to bind less Trf.

**Figure 2:**
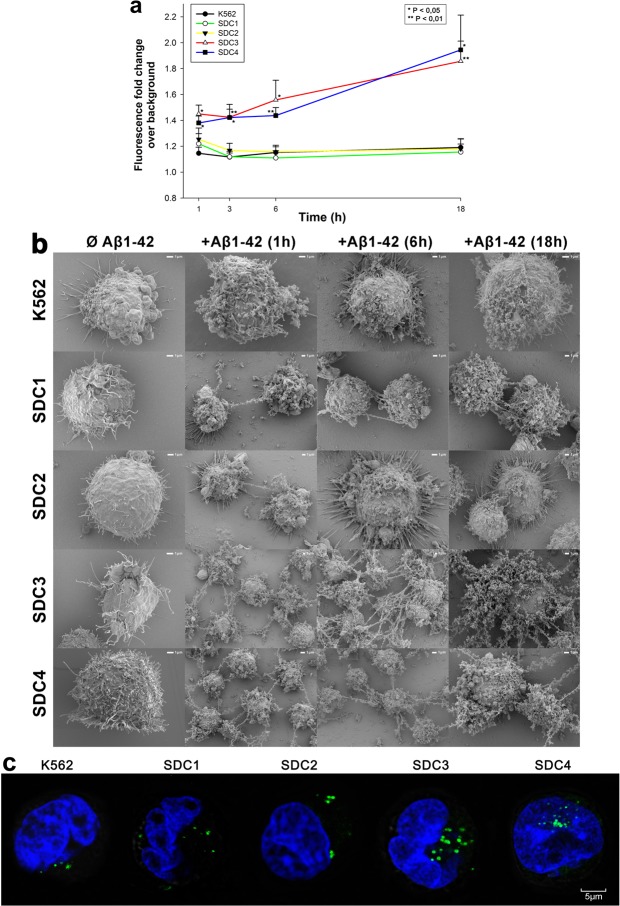
Effects of SDCs on Aβ1–42 fibrillation. WT K562 cells and SDC transfectants were incubated with Aβ1–42 at a concentration of 5 μM for 1 h to 18 h at 37 °C. (**a**) After 1, 3, 6 and 18 h of incubation, the cells were treated with ThT at a concentration of 15 μM for 10 mins and fluorescence was measured. Amyloid fluorescence is expressed as fold change over background ThT fluorescence. The bars represent mean ± SEM of four independent experiments. Statistical significance vs Aβ1–42-treated WT K562 cells was assessed by analysis of variance (ANOVA). *p < 0.05 vs Aβ1–42-treated WT K562 cells; **p < 0.01 vs Aβ1–42-treated WT K562 cells. (**b**) Scanning electron microscope visualization of WT K562 cells and SDC transfectants treated with Aβ1–42 at a concentration of 5 μM for 1, 6 or 18 h. Representative images of three independent experiments are shown. Scale bar = 1 μm. (**c**) CLSM visualization of ThT labeled, intracellular Aβ1–42 fibrils in WT K562 cells and SDC transfectants. Representative images of three independent experiments are shown. Scale bar = 5 μm.

After assessing uptake kinetics of Aβ1–42, we moved on to study the fibrillation of the peptide. It is widely established, that polyanions, like heparin and HS facilitates the fibrillation of Aβ1–42^34,67,68^. After demonstrating SDCs’ contribution to Aβ1–42 uptake, we also analyzed SDCs’ effect on Aβ1–42 fibrillation. ThT fluorescence studies quantitatively showed that SDCs, especially SDC3 and SDC4 significantly (p < 0.05) facilitate the fibrillation of Aβ1–42 (Fig. 2a). Compared to WT K562 cells with relatively low surface HS expression, SDC3 and SDC4 induce the fibrillation of Aβ1–42 even after 1 h of incubation, as shown by increased ThT fluorescence of Aβ1–42-treated SDC3 and 4 transfectants. Simultaneous scanning electron microscopy studies also confirmed the fibrillation triggering effect of SDCs - especially SDC3 and SDC4 - on surface attached Aβ1–42 (Fig. 2b). Thus scanning electron microscopy also showed increased number of fibrillar Aβ1–42 assemblies on SDC3 and SDC4 overexpressing cells, even after 1 h incubation. Confocal microscopy studies then revealed ThT labeled fibrillar structures inside of Aβ1–42 treated cells, especially in SDC3 and SDC4 transfectants, suggesting while triggering Aβ1–42 fibrillation, SDCs also mediate the intracellular translocation of Aβ1–42 oligomers (Fig. 2c).

**Figure 3:**
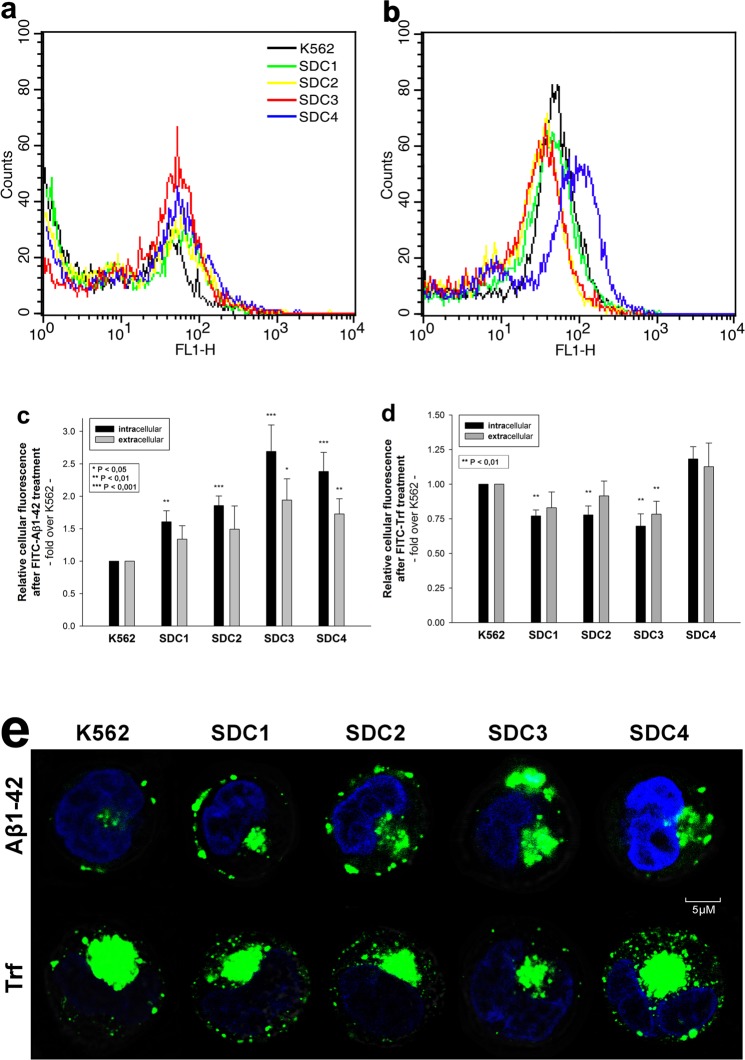
Cellular uptake of Aβ1–42 and Trf into WT K562 cells and SDC transfectants. WT K562 cells and SDC transfectants were incubated with FITC-labeled Aβ1–42 or Trf for 18 h at 37 °C. Cellular uptake of Aβ1–42 was then analyzed with flow cytometry and confocal microscopy. (**a**,**b**) Flow cytometry histograms showing intracellular fluorescence WT K562 cells and SDC transfectants, following 18 h incubation with Aβ1–42 and Trf, respectively. (**c**,**d**) Detected cellular fluorescence intensities were normalized to FITC-Aβ1–42 or FITC-Trf-treated WT K562 cells as standards. The bars represent mean ± SEM of nine independent experiments. Statistical significance vs standards (FITC-Aβ1–42 or FITC-Trf-treated WT K562 cells) was assessed by analysis of variance (ANOVA). *p < 0.05 vs standards; **p < 0.01 vs standards; ***p < 0.001 vs standards. (**e**) CLSM visualization of FITC-Aβ1–42 or FITC-Trf uptake into K562 cells and SDC transfectants. Nuclei of cells were stained with DAPI. Representative images of three independent experiments are shown. Scale bar = 5 μm.

Considering the results of the initial uptake kinetics and fibrillation studies, we moved on to analyze the cellular internalization of Aβ1–42 at 18 h of incubation, when differences in Aβ1–42 uptake and fibrillation was the greatest between SDC transfectants and WT K562 cells. Compared to WT K562 cells, all SDC transfectants exhibited higher uptake of Aβ1–42 at 18 h (p < 0.01). Among SDC transfectants, the neuron specific SDC3 internalized Aβ1–42 the most and Trf the least (Fig. 3a–d). Thus the previously observed difference in Aβ1–42 and Trf uptake (along with cellular attachment) became indeed obvious: while SDC1, 2 and 3 all increased internalization of Aβ1–42, cellular uptake of Trf, the marker of clathrin-mediated endocytosis were reduced in all these SDC transfectants (p < 0.01), suggesting that in case of SDC1, 2 and 3 transfectants, the internalization of Aβ1–42 and Trf occur through different routes. Compared to WT K562 cells, extracellular attachment of Aβ1–42 was also increased on SDC3 and 4 transfectants (p < 0.05 and p < 0.01, respectively), while in case of Trf, extracellular attachment to SDC3 transfectants was significantly (p < 0.01) reduced (Fig. 3c,d). Microscopic visualization demonstrated similar results as Aβ1–42-treated SDC transfectants exhibited higher intracellular fluorescent signals (Fig. 3e). On SDC transfectants, significant amount of fluorescence also arose from externally bound FITC-Aβ1–42. Trf treated cells on the other hand, exhibited fluorescent vesicle-like intracytoplasmic structures, a characteristic feature of clathrin-mediated endocytosis, but lacked surface attached aggregate-like structures observed on Aβ1–42-treated SDC transfectants (Fig. 3e).

**Figure 4:**
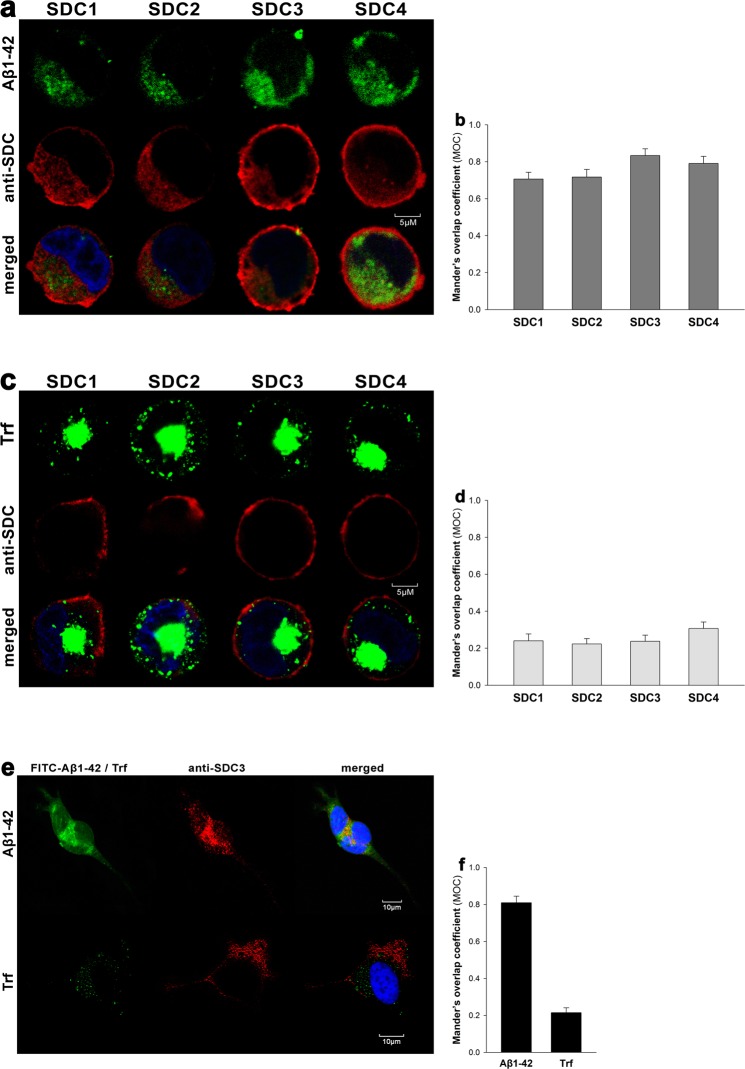
Colocalization of Aβ1–42 and SDCs. SDC transfectants or WT SH-SY5Y cells were treated with either FITC-Aβ1–42 or FITC-Trf for 18 h at 37 °C. After incubation, the cells were permeabilized and treated with the respective APC-labeled SDC antibody. Nuclei of cells were stained with DAPI and cellular uptake was then analyzed with CLSM. (**a**,**c**) CLSM images of SDC transfectants treated either FITC-Aβ1–42 (**a**) or FITC-Trf (**c**) and respective APC-labeled SDC antibody. Representative images of three independent experiments are shown. Scale bar = 5 μm. (**b**,**d**) Mander’s overlap coefficient (MOC) ± SEM for the overlap of SDCs with FITC-Aβ1–42 (**b**) or FITC-Trf (**d**) was calculated by analysis of 21 images with ~7 cells in each image (from three separate samples). (**e**) CLSM images of WT SH-SY5Y cells treated with FITC-Aβ1–42 or FITC-Trf, along with APC-labeled SDC3 antibody. Representative images of three independent experiments are shown. Scale bar = 10 μm. (**f**) Mander’s overlap coefficient (MOC) ± SEM for the overlap of SDC3 with FITC-Aβ1–42 or FITC-Trf was calculated by analysis of 21 images with ~7 cells in each image (from three separate samples)

CLSM colocalization studies showed strong intracellular colocalization of SDCs with Aβ1–42 (the Mander’s overlap coefficients [MOC] between Aβ1–42 and SDCs were all greater than 0.7, referring to strong colocalization), suggesting the common intracellular pathway SDCs and Aβ1–42 follow during cellular entry (Fig. 4a,b). It is worth noting that like neurons, SH-SY5Y neuroblastoma cells endogenously express SDC3 (Supplementary Fig. S4) and colocalization of SDC3 with Aβ1–42 could be also demonstrated in the SH-SY5Y cell-line (MOC = 0.81 ± 0.39; Fig. 4e,f). Co-immunoprecipitation on Aβ1–42 treated SDC transfectants and WT SH-SY5Y cells also confirmed that Aβ1–42 binds SDC3 (Supplementary Fig. S5). Unlike Aβ1–42, Trf did not exhibit colocalization with any SDCs after 18 h of incubation (MOC ≈ 0.25; Fig. 4c,d and f).

### SDC-mediated uptake of Aβ1–42 occurs through membrane microdomains

**Figure 5:**
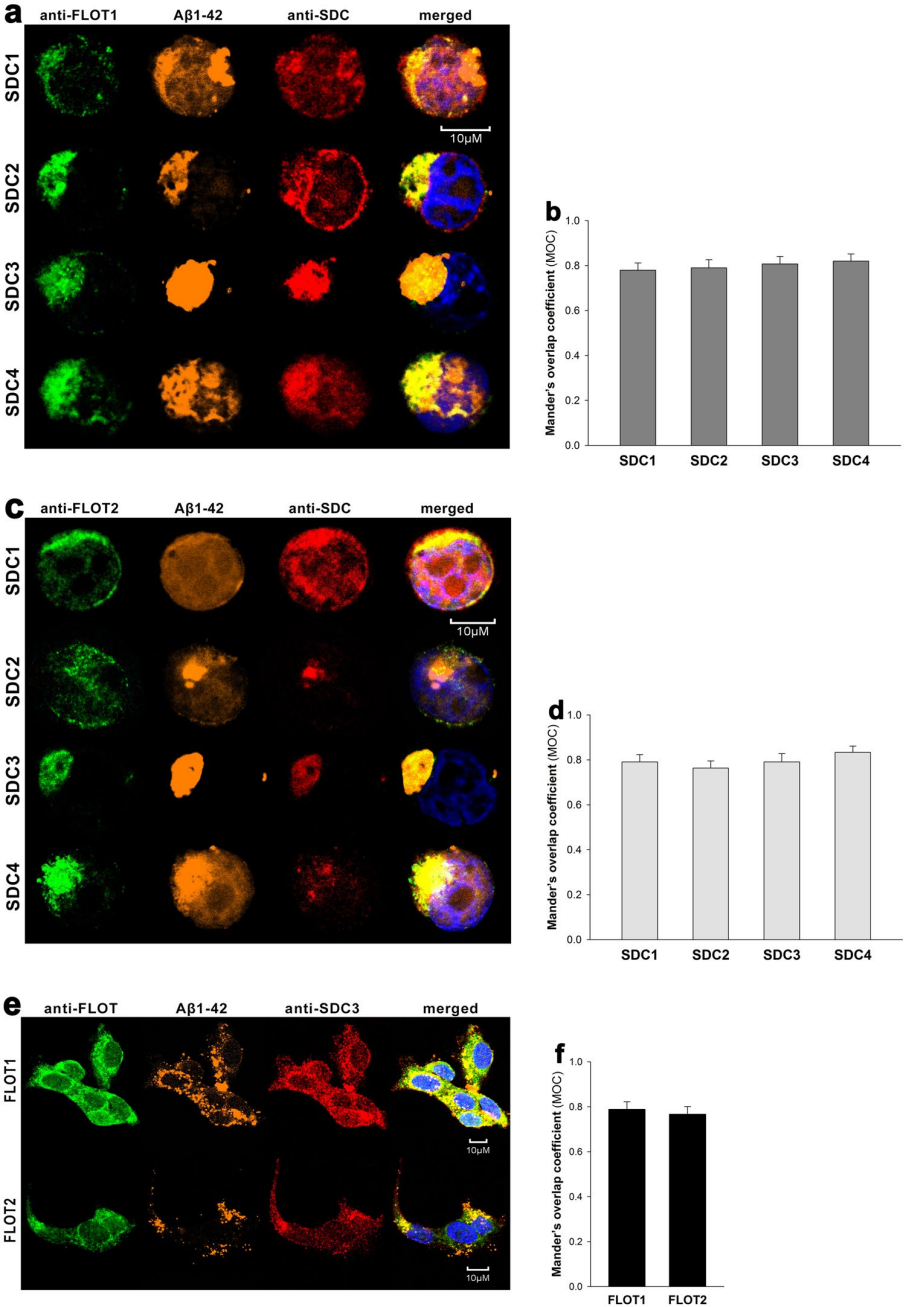
Colocalization of SDCs, Aβ1–42 and flotillins. SDC transfectants and WT SH-SY5Y cells were treated with 5-TAMRA-labeled Aβ1–42 for 18 h at 37 °C. After incubation, the cells were permeabilized and treated with the respective APC-labeled SDC antibody, along with either flotillin 1 (FLOT1) or flotillin 2 (FLOT2) antibodies (both Alexa Fluor 488-labeled). Nuclei of cells were stained with DAPI and cellular uptake was then analyzed with CLSM. Representative images of three independent experiments are shown. (**a**,**c**) CLSM images of SDC transfectants treated with FITC-Aβ1–42, one of the anti-flotillin antibodies and a respective APC-labeled SDC antibody. (**b**,**d**) Mander’s overlap coefficient (MOC) ± SEM for the overlap of SDCs with either FLOT1 or FLOT2 was calculated by analysis of 21 images with ~7 cells in each image (from three separate samples). (**e**) CLSM images of WT SH-SY5Y cells treated with 5-TAMRA-labeled Aβ1–42, APC-labeled SDC3 antibody and FLOT1 or FLOT2 antibodies. Scale bar = 10 μm. (**f**) Mander’s overlap coefficient (MOC) ± SEM for the overlap of SDC3 with either FLOT1 or FLOT2 was calculated by analysis of 21 images with ~7 cells in each image (from three separate samples).

It has been reported, that HSPGs are endocytosed through a clathrin- and caveolin-independent, but flotillin-dependent route^69^. The flotillin family of membrane proteins, namely flotillin 1 (FLOT1) and flotillin 2 (FLOT2) define specific microdomains in the plasma membrane. Flotillins have been long considered markers of lipid rafts because they are detergent insoluble and float in sucrose density gradients. CLSM studies showed the intracellular colocalization of both flotillins with Aβ1–42 and SDCs in SDC transfectants, while WT SH-SY5Y cells also exhibited intraneuronal colocalization of flotillins with Aβ1–42 and SDC3, suggesting that SDC-dependent uptake of Aβ1–42 occur through lipid rafts (Fig. 5a–f). With affinity-based proteomics FLOT1 and -2 were detected in a pull-down experiment of SDC4 with 43/210/79.9% and 41/203/72.7% frequency (number of unique peptide/peptide count/coverage, respectively), thus confirming the findings of the CLSM colocalization studies (Supplementary Table S1).

### Effect of SDC domains on Aβ1–42 uptake and fibrillation

**Figure 6:**
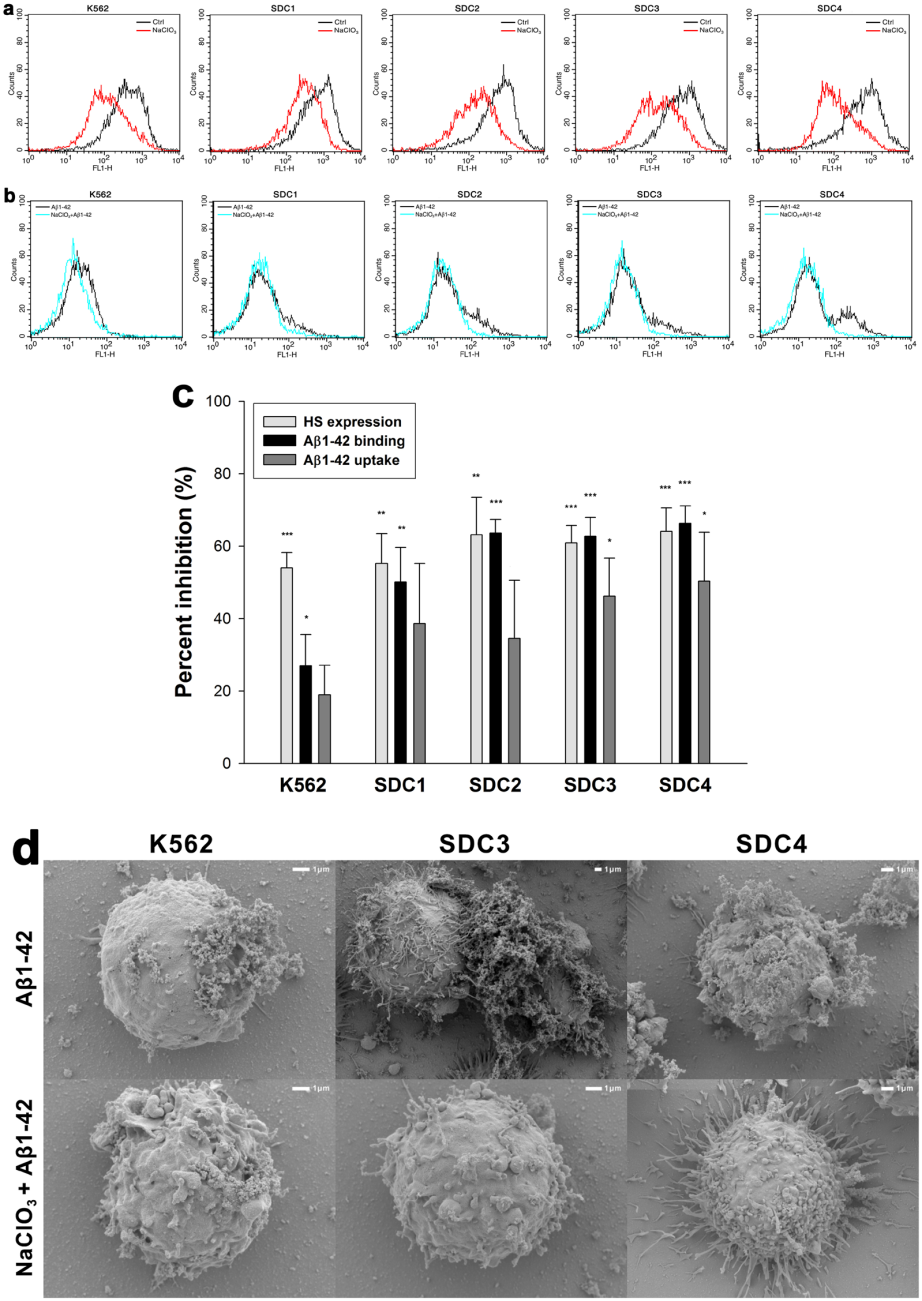
Effect of undersulfation on Aß1–42 uptake and binding. WT K562 cells and SDC transfectants were incubated with sodium chlorate (NaClO3) prior to Aβ1–42 treatment. Effect of NaClO3 on HS expression, along with Aβ1–42 and attachment were assessed with flow cytometry. (**a**) Flow cytometry histograms showing HS expression of WT K562 cells or SDC transfectants after NaClO3 treatment. (**b**) Flow cytometry histograms representing intracellular fluorescence of FITC-Aβ1–42-treated cells preincubated with or without NaClO3. (**c**) The effect of NaClO3 were expressed as percent inhibition, calculated with the following formula: [(X − Y)/X] × 100, where X is the fluorescence intensity obtained on cells treated with Aβ1–42 in the absence of the NaClO3 and Y is the fluorescence intensity obtained on cells treated with either Aβ1–42 in the presence of the NaClO3. The bars represent mean ± SEM of four independent experiments. Statistical significance vs controls untreated with NaClO3 was assessed by analysis of variance (ANOVA). *p < 0.05 vs standards; **p < 0.01 vs standards; ***p < 0.001 vs standards. (**d**) Scanning electron microscope visualization of cellular surface of Aβ1–42-treated WT K562 cells and SDC3, SDC4 transfectants, preincubated with or without NaClO3. Representative images of three independent experiments are shown. Scale bar = 1 μm.

**Figure 7:**
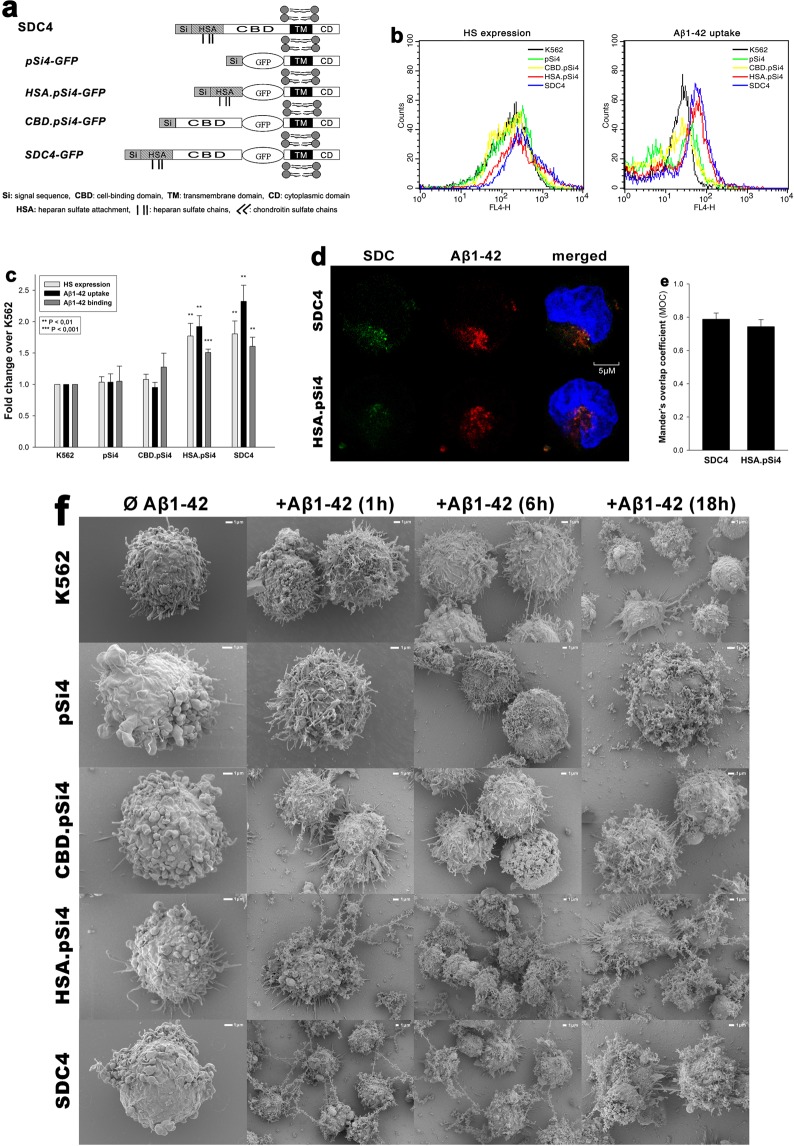
Contribution of various parts of the SDC4 ectodomain to Aβ1–42 uptake. WT K562 cells and SDC4 mutants were treated with Aβ1–42 at a concentration of 5 μM at 37 °C. (**a**) Schematic representation of SDC4 deletion mutants used in the study. (**b**) Flow cytometry histograms representing HS expression and intracellular fluorescence of WT K562 cells and SDC4 mutants treated with FITC-Aβ1–42 for 18 h. (**c**) Results of flow cytometric measurements. Detected fluorescence intensities are normalized to FITC-Aβ1–42-treated WT K562 cells as standards. The bars represent mean ± SEM of four independent experiments. Statistical significance vs FITC-Aβ1–42-treated WT K562 cells was assessed by analysis of variance (ANOVA). **p < 0.01 vs standards; ***p < 0.001 vs standards. (**d**) CLSM visualization of SDC4 or HSA.pSi4 transfectants treated with FITC-Aβ1–42 for 18 h. Scale bar = 5 μm. (**e**) Mander’s overlap coefficient (MOC) ± SEM for the overlap of Aβ1–42 with either SDC4 or HAS.pSi4 was calculated by analysis of 21 images with ~7 cells in each image (from three separate samples). (**f**) Scanning electron microscope visualization of cellular surface of Aβ1–42-treated WT K562 cells and SDC4 mutants. Representative images of three independent experiments are shown. Scale bar = 1 μm.

To understand the involvement of SDC domains in the interaction with Aβ1–42, we set up studies where various parts of the SDC ectodomain were affected. First we induced undersulfation of SDCs’ HS chains with sodium chlorate (NaClO3), a well-known inhibitor of protein sulfation^70,71^. Promoting undersulfation of SDCs with NaClO3 exerted an evident and significant (p < 0.05) inhibition of Aβ1–42 attachment in all cells lines, however the inhibitory effect on uptake was only significant (p < 0.05) in case of SDC3 and 4 transfectants (especially SDC3 and 4) than in WT K562 cells (Fig. 6c). Scanning electron microscopy studies also confirmed the inhibitory effect of undersulfation as fibrillary assemblies of Aβ1–42 were reduced due to NaClO3 treatment (Fig. 6d). Next we created structural mutants of SDC4, an isoform that exhibited high uptake efficacy of Aβ1–42 in the previous uptake assays. As SDC4 has a specific cell-binding-domain (CBD) that mediates cell-to-cell attachment, we also addressed the potential contribution of CBD to interaction with Aβ1–42. Thus a number structural mutants of SDC4 were created, including the CBD.pSi4 made of the CBD and the secretion signal sequence (Si), but lacking any HS chains. HSA.pSi4 mutants on the other hand lack CBD, but contain the HS attachment site (HSA) with the HS chains, while pSi4 mutants possess a truncated extracellular domain made of only the Si of SDC4 (Fig. 7a). All of the above mentioned SDC4 mutants, along with one coding WT SDC4, were tagged with GFP at the juxtamembrane region and expressed in K562 cells. Clones with equal extent of GFP, hence SDC expression were then selected with flow cytometry and – along with WT K562 cells as controls – treated with Aβ1–42 for 18 h at 37 °C. Cellular uptake and attachment was then analyzed with flow cytometry, as well as confocal and scanning electron microscopy. Compared to K562 cells, WT SDC4 transfectants exhibited the most significant (p < 0.01) increase in Aβ1–42 uptake (Fig. 7b,c). Mutants lacking HS chains (i.e. pSi4 or CBD.pSi4) did not induce any increase in Aβ1–42 uptake, suggesting that neither the signal sequence nor the CBD domain interacts significantly with Aβ1–42. Mutants with truncated ectodomain made of only the HSA with the HS chains on the other hand increased Aβ1–42 internalization at an extent similar to SDC4 transfectants, showing the paramount importance of polyanionic HS chains in the interactions with Aβ1–42. Similarly to SDC4 transfectants, microscopic visualization also confirmed the intracellular colocalization of HSA.pSi4 with Aβ1–42 (MOC = 0.74), suggesting that Aβ1–42 enters the cells attached to HS chains of SDC4 (Fig. 7d,e). Scanning electron microscopy then demonstrated a less pronounced fibrillary assemblies of Aβ1–42 on SDC4 mutants lacking HS chains (Fig. 7e).

**Figure 8:**
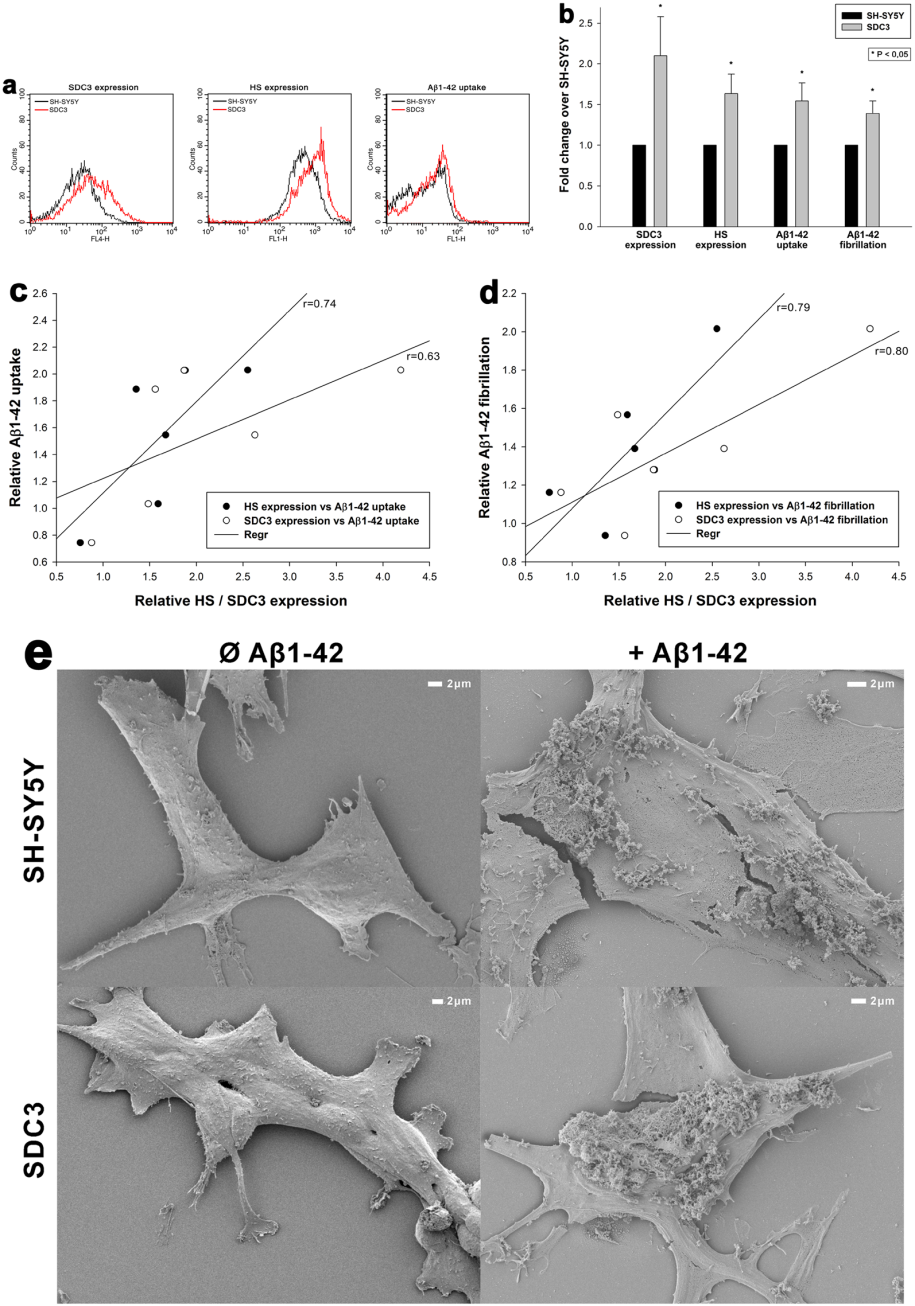
Effect of SDC3 on Aβ1–42 uptake and fibrillation in neurons. SDC3 transfectants created in SH-SY5Y cells were selected by measuring SDC3 expression with flow cytometry using APC-labeled anti-SDC3 antibody. HS expression of SDC3 transfectants, along with WT SH-SY5Y, was also measured with flow cytometry using anti-HS antibody. SDC3 transfectants and WT SH-SY5Y cells were treated with Aβ1–42 (with or without FITC label) at a concentration of 5 μM at 37 °C. Cells incubated with Aβ1–42 for 18 h were then processed for uptake, fibrillation studies and scanning electron microscopy. (**a**) Flow cytometry histograms representing SDC3, HS expression levels and intracellular fluorescence of Aβ1–42-treated WT SH-SY5Y cells and SDC3 transfectants. (**b**) Fold change in SDC3 and HS expression, along with Aβ1–42 uptake and fibrillation following SDC3 overexpression. The bars represent mean ± SEM of six independent experiments. Statistical significance vs Aβ1–42-treated WT SH-SY5Y cells (standards) was assessed by analysis of variance (ANOVA). *p < 0.05 vs Aβ1–42-treated WT SH-SY5Y cells (standards). (**c**,**d**) Linear regression between HS or SDC3 expression and Aβ1–42 uptake or fibrillation. (**e**) Scanning electron microscopy visualization of Aβ1–42 attachment and fibrillation on WT SH-SY5Y cells and SDC3 mutants. Representative images of three independent experiments are shown. Scale bar = 2 μm.

After assessing SDCs’ contribution to Aβ1–42 fibrillation and cellular entry in SDC overexpressing K562 cells, we also analyzed the contribution of SDC3 to Aβ1–42 fibrillation and uptake in the more complex SH-SY5Y neuronal model cell line. Thus SDC3 overexpressing SH-SY5Y cells were created and the effect of SDC3 overexpression on Aβ1–42 fibrillation and uptake cellular was studied. In SH-SY5Y cells, SDC3 overexpression resulted in increased HS expression, along with increased cellular uptake and fibrillation of Aβ1–42 (Fig. 8a–e). Thus a very definite correlation was found between SDC3 overexpression and Aβ1–42 uptake (r = 0.63) or fibrillation (r = 0.80) in SH-SY5Y neurons (Fig. 8c,d). HS expression also exhibited high correlation with Aβ1–42 uptake (r = 0.74) and fibrillation (r = 0.79), further confirming the roles of HS chains in interactions with Aβ1–42 (Fig. 8c,d).

## Discussion

There is compelling evidence that Aβ1–42, rendered in β-sheet dominated senile plaques, is a hallmark of Alzheimer’s disease (AD) in the brain of late-life dementia patients. Despite of growing criticism on the Aβ hypothesis, new lines of evidence support the concept that an imbalance between production and clearance of Aβ1–42 peptides is a very early, often initiating factor in Alzheimer’s disease (AD)^72^. Intraneuronal accumulation of Aβ aggregates due to Aβ re-uptake has been observed to precede extracellular plaque formation in AD^18,19,73,74^. As detailed exploration of Aβ1–42 endocytosis contributes to the understanding of cellular pathomechanism of neurodegeneration, extensive studies have been conducted and revealed the macropinocytic nature of Aβ1–42 cellular uptake and the involvement of cell surface HSPGs^13,67,75–77^. Meanwhile, our research group has been exploiting the SDC-mediated macropinocytic delivery of peptides that enables the efficient intracellular transport of bioactive macromolecules^56,58^. Considering the similarities on the cellular uptake of CPPs and Aβ1–42, we investigated the cellular uptake of Aβ1–42 in cellular models specific to various SDC isoforms. Our studies explored the exact contribution of each SDC isoform to Aβ1–42 internalization: overexpression of all SDCs increased the uptake of Aβ1–42, however the increase was fairly different among the various SDC isoforms. Thus the neuron specific SDC3 increased Aβ1–42 uptake from the earliest time points and SDC4, the universally expressed isoform of the family, increased Aβ1–42 uptake at a pace similar to SDC3. Contrary to SDC3 and 4, SDC1- and 2-mediated internalization of Aβ1–42 was markedly slower. SDC-mediated cellular entry of Aβ1–42 via SDCs was shown to occur through lipid rafts and attachment to polyanionic HS chains. Among the various parts of the SDC ectodomain, the HS chains proved to have a major role in interacting with Aβ1–42. As SDC transfectants applied expressed equal amount of HS, the observed difference between the various SDCs in Aβ1–42 fibrillation could be attributed to the structural differences in HS chains of the distinct SDC isoforms. Also the different SDC isoforms interact with different set of intracellular signaling molecules and that could also contribute to the observed difference in Aβ1–42 uptake.

The high affinity of Aβ1–42 towards the neuron specific SDC3 isoform, along with the fast and marked uptake into SDC3 transfectants, could account for the tendency of Aβ1–42 affecting neurons: SDC3 on neurons would bind and internalize Aβ1–42 even before it could be cleared off the extracellular space by phagocytic cells. The observation SDC3 overexpression triggers Aβ1–42 fibrillation suggest that significant part of Aβ1–42 enter SDC3 expressing cells in fibrillar form, thus implying the negative effect of SDC3 overexpression on neurons, as observed by Liu *et al*. in their present study on postmortem human AD brains^11^. The decreased uptake of Trf, the marker of clathrin-dependent endocytosis, into SDC3 transfectants also implies that SDC3 overexpression affects the classical endocytosis, thus clearance of Aβ1–42, leading to intraneuronal accumulation of oligomeric Aβ1–42 via lipid-raft dependent, alternative endocytic pathways.

Overall, our data represents SDCs, especially SDC3 and SDC4 as a preferred cellular targets for Aβ1–42 to enter the cells. Considering significant overexpression of SDC3 and SDC4 in human AD brains, our SDC dependent cellular assays provided results relevant to human conditions and revealed unknown details of SDC-mediated endocytosis and fibrillation of Aβ1–42. The observation that SDC3 overexpression, regardless of cell type, can contribute to fundamental biological processes (i.e. uptake and fibrillation) related to Aβ1–42 pathology, highlights SDCs, especially SDC3, the neuron specific member of the SDC family with increased expression in AD brains as important players in the pathophysiologic events of neurodegeneration.

## Methods

### Peptides

Fluorescently labeled (FITC, 5-TAMRA and HiLyte™ Fluor 647) Aβ1–42 was purchased from BACHEM and AnaSpec, respectively. FITC-labeled human Trf, the marker of clathrin-mediated endocytosis was purchased from Thermo Fisher Scientific.

### SDC constructs, cell culture and transfection

Full-length SDC1-4 and SDC4 deletion mutants were amplified and subcloned in the mammalian expression plasmid obtained from Clontech (pcDNA3, pEGFP). Structural SDC4 mutants were constructed by inserting green fluorescent protein (GFP) into the juxtamembrane region of the extracellular segment. The signals were kept in all cases to orient the proteins into the membrane. Human SDC DNA constructs were prepared as described above and transfected into K562 cells with DMRIE-C (Invitrogen) to establish stable cell lines permanently expressing human SDCs or SDC4 structural mutants^56^. Transfections were carried out according to the recommendations of the manufacturer. After 24 h, cells were incubated with selection medium, containing 0.4 mg/ml G418 (Sigma). Selection medium was changed every second day. After 2 weeks, G418-resistant colonies were analyzed for the SDC expression with flow cytometry using APC-labeled antibodies specific for the given SDC isoforms (R&D Systems). In case of GFP-tagged SDC4 mutants, expression was analyzed with flow cytometry measuring fluorescence intensities of the GFP tags. Thus colonies exhibiting marked SDC expression were chosen for further studies. The erythroleukemia cell line K562 and its SDC clones were grown as a suspension culture in DMEM/F12 medium (Thermo Fischer Scientific) supplemented with 10% fetal calf serum (FCS; Gibco) at 37 °C in a humified 5% CO_2_ containing air environment. In case of SDC3 overexpressing SH-SY5Y cells, transfection and selection of SDC3 overexpressing clones were carried out as described above. SDC3 overexpressing SH-SY5Y clones, along with WT SH-SY5Y cells were then grown in Advanced MEM medium (Thermo Fischer Scientific) supplemented with 10% FCS (Gibco) at 37 °C in a humified 5% CO2 containing air environment.

### Flow cytometry analysis of HS and CS expression

As HS was shown to attach Aβ1–42, HS expression of wild-type (WT) K562 cells, SDC transfectants and SH-SY5Y cells were measured with flow cytometry by using anti-human HS antibody (10E4 epitope [Amsbio]) and FITC- or Alexa 647-labeled goat anti-mouse IgG (Sigma) according to the manufacturers’ protocols. SDC transfectants with almost equal amount of HS expression were selected for further uptake studies. SDC transfectants were also assessed for CS expression, using monoclonal anti-CS primary antibody (Clone CS-56, Sigma) and FITC-labeled goat anti-mouse IgG (Sigma) according to the manufacturers’ protocols.

### Flow cytometry analysis of cellular uptake

WT K562 and SH-SY5Y cells, SDC transfectants, along with SDC4 structural mutants were utilized to quantify internalization of fluorescently labeled Aβ1–42 or Trf. Briefly, 6 × 10^5^ cells/ml in DMEM/F12 medium (with 10% FCS) were incubated with fluorescently labeled (FITC-, or in the case of SDC4 mutants, HiLyte™ Fluor 647) Aβ1–42 or Trf (at a concentration of 5 μM and 25 μg/ml, respectively), for various amounts of time (1, 3, 6 and 18 h) at 37 °C. After incubation the cells were washed twice in ice cold PBS and progressed towards flow cytometry. In the case of the FITC-Aβ1–42 and Trf-treated cells, after incubation and washing, the cells (WT K562, SH-SY5Y and SDC transfectants) were resuspended in 0.5 ml of physiological saline. Equal volumes of this suspension and a stock solution of trypan blue (Merck KGaA; 500 μg/ml dissolved in ice-cold 0.1 M citrate buffer at pH 4.0) were allowed to mix for 1 min before the flow cytometric analyses. In this way, sample pH was lowered to pH 4.0, thereby optimizing the quenching effect of trypan blue to quench the extracellular fluorescence of surface bound fluorescent proteins^66^. In the case of the SDC4 mutants treated with HiLyte™ Fluor 647-labeled Aβ1–42, extracellular fluorescence of surface attached peptides was removed by trypsinization according to the method described by Nakase *et al*.^78^. Cellular uptake and attachment was then measured by flow cytometry using a FACScan (Becton Dickinson). Cellular attachment was calculated by subtracting intracellular fluorescence (i.e. those quenched with trypan blue or trypsin) from measures of overall fluorescence. A minimum of 10,000 events per sample was analyzed. Viability of cells was determined by appropriate gating in a forward-scatter-against-side-scatter plot to exclude dead cells and debris.

### Microscopic visualization of cellular uptake

Internalization of the fluorescently labeled (either FITC, 5-TAMRA or HiLyte™ Fluor 647) Aβ1–42 or Trf was visualized by confocal laser scanning microscopy (CLSM). WT SH-SY5Y and WT K562 cells, along with SDC transfectants and SDC4 mutants were grown on poly-D-lysine-coated glass-bottom 35-mm culture dishes (MatTek Corp.). After 24 h, the cells were preincubated in DMEM/F12 medium (Supplemented with 10% FCS) at 37 °C for 30 min before incubation with the fluorescently labeled Aβ1–42 or Trf at a concentration of 5 μM and 25 μg/ml, respectively. After incubation, the cells were rinsed two times with ice-cold PBS, fixed in 4% paraformaldehyde (Sigma) and nuclei were stained with DAPI (1:5000, Sigma) for 5 min. For colocalization studies, after fixation, the cell membranes were permeabilized (1% Triton X-100), followed by 1 h of treatment with APC-labeled SDC antibodies (1:100) with or without either of the Alexa Fluor® 488-labeled flotillin antibodies (flotillin-1 or 2, all Santa Cruz Biotech). The samples were then rinsed three times with PBS containing 1% goat serum and 0.1% Triton X-100, then stained with DAPI (1:5000) for 5 min, washed three times with PBS and embedded in Fluoromount G (SouthernBiotech). Distribution of fluorescence was then analyzed on an Olympus FV1000 confocal laser scanning microscope equipped with three lasers. A laser diode (excitation, 405 nm) and a band-pass filter (420–480 nm) were used to capture the signal recorded as blue; an argon laser (excitation, 488 nm) and a bandpass filter (505–530 nm) were used to capture the signal recorded as green; and finally, a helium/neon laser (excitation, 543 nm) and a band-pass filter (550–625 nm) were used to capture the signal recorded as red. Sections presented were taken approximately at the mid-height level of the cells. Photomultiplier gain and laser power were identical within each experiments. FV10-ASW software was used for image acquisition by confocal microscopy. For visualizing internalization of oligomeric Aβ1–42, WT K562 cells and SDC transfectants grown on poly-D-lysine-coated glass-bottom 35-mm culture dishes were incubated with Aβ1–42 (BACHEM) at a concentration of 5 µM (in DMEM/F-12 without Phenol Red) at 37 °C for 18 h, then treated with Thioflavin T (ThT, Sigma) at a concentration of 25 µM for 10 min at 37 °C and rinsed two times with ice-cold PBS. After fixation in 4% paraformaldehyde (Sigma), nuclei were stained with DAPI (1:5000) for 5 min, the after three washing with PBS, the samples were embedded in Fluoromount G and distribution of fluorescence was analyzed on an Olympus FV1000 confocal laser scanning as described above. For colocalisation analyses (SDCs with Aβ1–42 or Trf; SDCs with flotillins, SDC3 with Aβ1–42 or flotillins) the images were analyzed in the ImageJ software (NIH, Bethesda, Maryland, US) with the plug-in JACoP28 as described by Wesén *et al*.^13^ 21 images (7 images per sample, experiment performed in triplicate) were analysed, and the data is presented as mean ± SEM.

### Co-immunoprecipitation experiments

Stable SDC3 transfectants or WT SH-SY5Y cells were incubated with or without FITC-labeled Aβ1–42 at a concentration for 18 h at 37 °C. After incubation the cells were washed twice with ice cold PBS and treated with cold Pierce IP lysis buffer. Then the cells were scrapped off to clean Eppendorf tubes, put on a low-speed rotating shaker for 15 min and centrifuged at 14,000 g for 15 min at 4 °C. The supernatant were then transferred to new tubes and combined with 5 µg of the human SDC3 antibody (R&D Systems). The antigen sample/SDC3 antibody mixture was then incubated for overnight at 4 °C with mixing. The antigen sample/SDC3 antibody mixture then were added to a 1,5 ml microcentrifuge tube containing pre-washed Pierce Protein A/G Magnetic Beads (Thermo Fisher Scientific) and after incubation at room temperature for 1 hour with mixing, the beads were then collected with a MagJET Separation Rack magnetic stand (Thermo Fisher Scientific) and supernatants were discarded. To eluate the antigen, 100 µl of SDS-PAGE reducing sample buffer were then added to the tubes and samples were heated at 96 °C for 10 minutes and the samples were proceeded to SDS-PAGE.

### Affinity-based proteomics of SDC4 interactions

To reveal the SDC4 protein interactome, whole cell extracts were prepared from stable transfectant of GFP-tagged SDC4, and then a GFP co-immunoprecipitation (GFP co-IP) assay was performed. Protein concentration of the whole cell extract (WCE) was determined by using Bradford Protein Assay (Bio-Rad, Hercules, California, USA), according to the manufacturer’s instructions. Total protein extracts (2 mg) were immunopurified (IP) using 40 µl anti-GFP antibody-coupled 50 nm superparamagnetic beads (µMACS GFP Isolation Kit, Miltenyi Biotec, Germany). The unbound material was removed by washing the beads with 2 ml (equal to 50x beads volume) detergent-free buffers as follows: three times with 1x TBS and once with 25 mM ABC(NH_4_HCO_3_) buffer. The immunopurified proteins were desalted after on-bead-digestion with trypsin (Promega, Germany)^79^. The LC-MS/MS analysis was performed by using a nanoflow RP-HPLC on-line coupled to a linear ion trap-Orbitrap (Orbitrap-Elite, Thermo Fisher Scientific, Germany) mass spectrometer as in a previous study with the following modification: the 20 most abundant, multiply charged ions were selected from each MS survey for MS/MS analysis^80^. Raw data were converted into peak lists using Proteome Discoverer (v 1.4, Thermo Fisher Scientific). First, we performed a search against the Swissprot and Uniprot databases, taking into consideration of the sequence of SDC4. Search parameters and acceptance criteria were set as previously published. Close homologues were only reported if at least three unique peptides matched to the protein. Spectral counting was used to estimate relative abundance of individual proteins in the samples: peptide counts of the individual proteins were normalized to the total number of peptide identifications in each sample^81^. Proteins (i) with reproducible detection (|log 2 fold-change| <0.67 between biological replicates), (ii) with at least two identified peptides, (iii) with at least 5% coverage and (iv) with a median-normalized protein binding affinity score above a previously defined cut-off value were considered as proteins that specifically associate with SDC4^82^.

### Thioflavin T binding assays

WT K562, SH-SY5Y cells and SDC transfectants seeded into black-sided, clear bottom 96-well microplates (Corning) at a density of 1,5 × 10^5^ cells/well in 100 µl of DMEM/F-12 (without Phenol Red) were exposed to Aβ1–42 at a concentration of 5 µM for various amounts of time (1, 3, 6 and 18 h) at 37 °C. After the incubation periods, Thioflavin T (ThT) was added to the Aβ1–42-treated cells at a concentration of 15 µM and after 10 min of incubation fluorescence was measured with Cytation™ 3 Multi-Mode reader (BioTek Instruments) using an excitation wavelength of 440 nm and an emission of 480 nm. Photomultiplier gain was set at 50. Fluorescence measurements are made from the bottom of the plate, with the top being sealed with an adhesive plate sealer to prevent evaporation. The fold change in ThT fluorescence intensity over background ThT signal was calculated by dividing the fluorescence intensity of the Aβ1–42-treated cells incubated with ThT by the respective fluorescence intensity of the ThT-incubated (same) cell line untreated with Aβ1–42^83^.

### Scanning electron microscopy of surface attachment and fibrillation of Aβ1–42

WT SH-SY5Y and WT K562 cells, along with SDC transfectants and SDC4 mutants were grown on poly-D-lysine-coated glass-bottom 35-mm culture dishes. After 24 h, the cells were preincubated in DMEM/F12 medium (supplemented with 10% FCS) at 37 °C for 30 min before incubation with Aβ1–42 at a concentration of 5 μM for 1, 6 or 18 h. Then the cells were rinsed two times with ice-cold PBS, then fixed in 2.5% glutaraldehyde and 0.15% alcian blue 8GX (Sigma) for 1 hour. After post-fixation in 1% OsO4 (Sigma) for 1 hour the samples were dehydrated in aqueous solutions of increasing ethanol concentrations, critical point dried, covered with 10 nm gold by a Quorum Q150T ES sputter and observed in a JEOL JSM-7100F/LV scanning electron microscope.

### Promoting undersulfation

To study the effect of proteoglycan sulfation, cells were incubated with 60 mM sodium chlorate (NaClO_3_; Sigma) for 48 h, the processed for the flow cytometry and microscopy studies as described above.

### Statistical analysis

Results are expressed as means ± standard error of the mean (SEM). Differences between experimental groups were evaluated by using one-way analysis of variance (ANOVA). Values of p < 0.05 were accepted as significant. Pearson’s correlation coefficient was calculated for correlation between the SDC3 or HS expression and Aβ1–42 uptake and fibrillation values of SH-SY5Y cells. For colocalisation analyses, the Mander’s overlap coefficient (MOC) was calculated as described by Wesen *et al*.^13^.

## Supplementary information

SUPPLEMENTARY INFO
