# Supplementary material for: Contribution of syndecans to cellular internalization and fibrillation of amyloid-β(1–42)
Source: Sci Rep. 2019 Feb 4;9:1393. doi: 10.1038/s41598-018-37476-9 (PMC6362000; doi:10.1038/s41598-018-37476-9)
Supplement: Supplementary file 1 — SUPPLEMENTARY INFO [file 41598_2018_37476_MOESM1_ESM.docx]

Supplementary Information

**Contribution of syndecans to cellular internalization and fibrillation of amyloid-β(1-42)**

**Tamás Letoha^1^*, Anett Hudák^1^, Erzsébet Kusz^1^, Aladár Pettkó-Szandtner^2^, Ildikó Domonkos^2^, Katalin Jósvay^2^, Martin Hofmann-Apitius^3^ and László Szilák^4^**

^1^Pharmacoidea Ltd., Szeged, H-6726, Hungary

^2^ Biological Research Center of the Hungarian Academy of Sciences, Szeged, H-6726, Hungary

^3^Fraunhofer Institute for Algorithms and Scientific Computing (SCAI), Sankt Augustin, 53754, Germany

^4^Szilak Laboratories, Bioinformatics and Molecule-Design, Szeged, H-6723, Hungary

*Corresponding author; e-mail: [tamas.letoha@pharmacoidea.eu](mailto:*tamas.letoha@pharmacoidea.eu), phone: +36 30 2577393

**
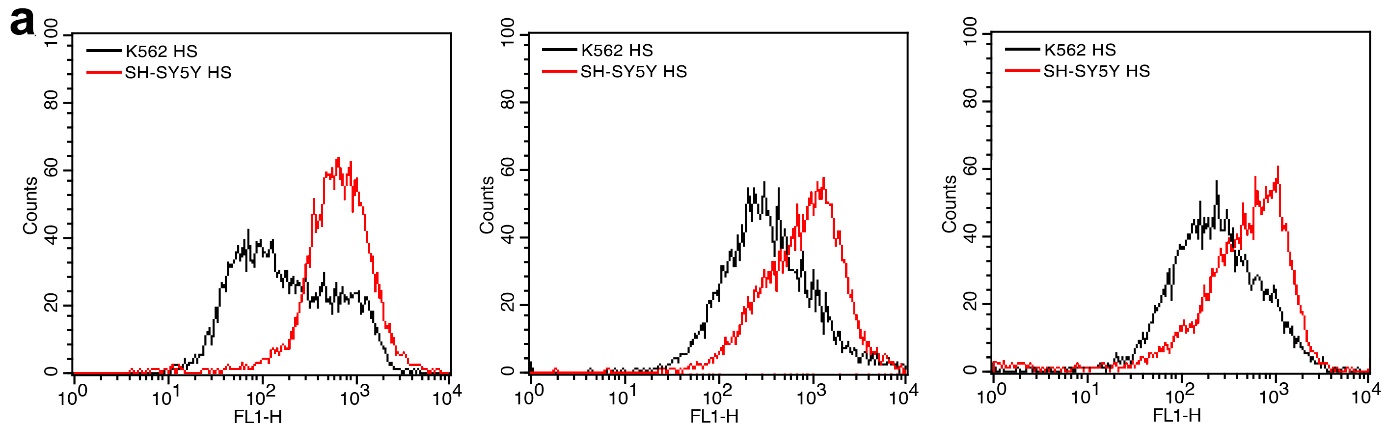
**

**
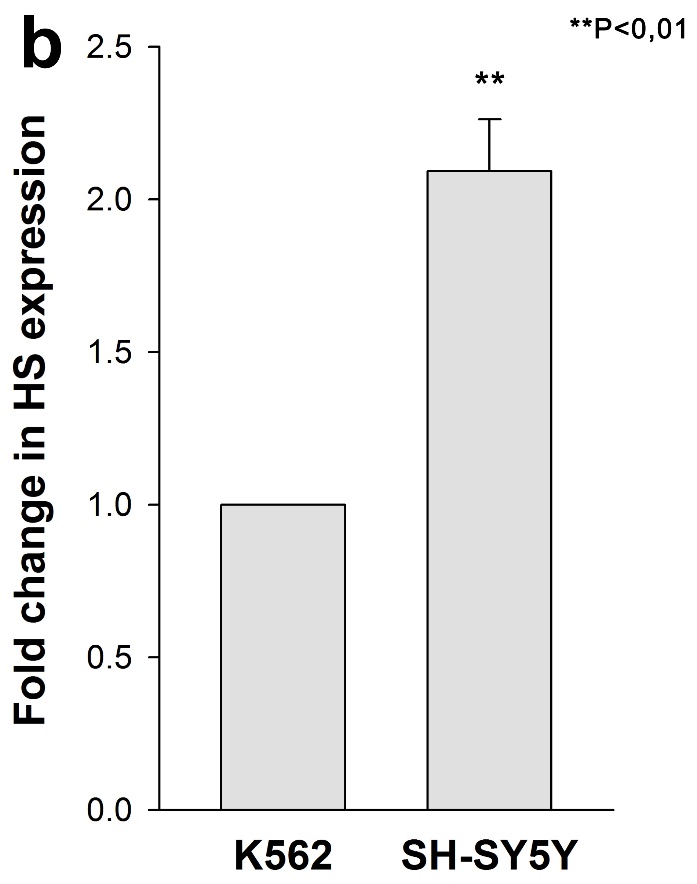
**

**Supplementary Fig. S1.** Relative HS expression of SH-SY5Y vs K562 cells. HS expression of K562 and SH-SY5Y cells was measured by flow cytometry using anti-HS antibody. **a**: Flow cytometry histograms showing the HS expression of SDC transfectants and K562 cells. **b**: Detected HS levels of were normalized to K562 cells as standards. The bars represent mean ± SEM of three independent experiments. Statistical significance vs K562 cells as standards was assessed by analysis of variance (ANOVA). **p<0.01 vs K562 cells.


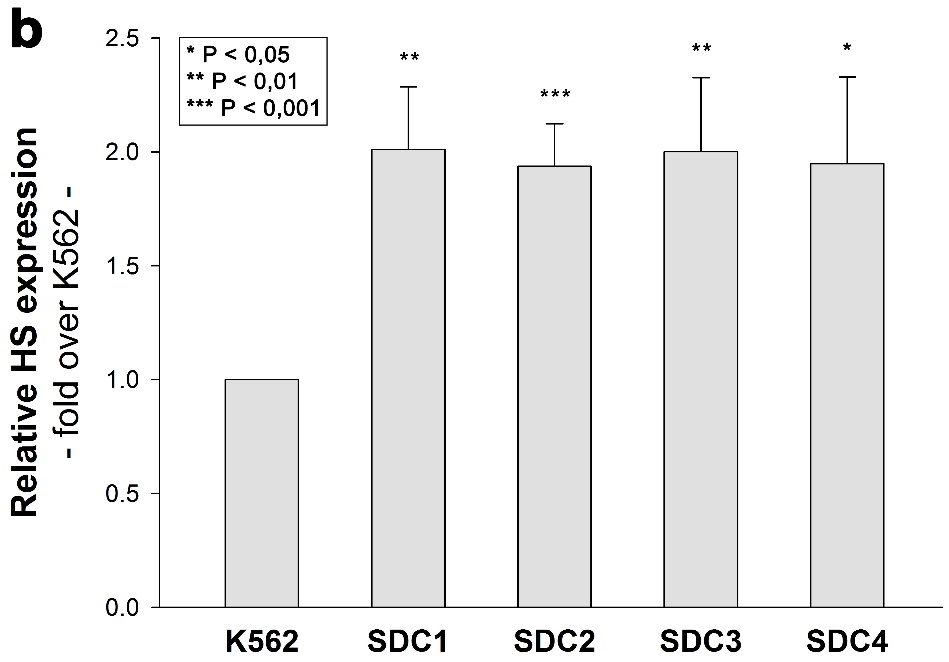


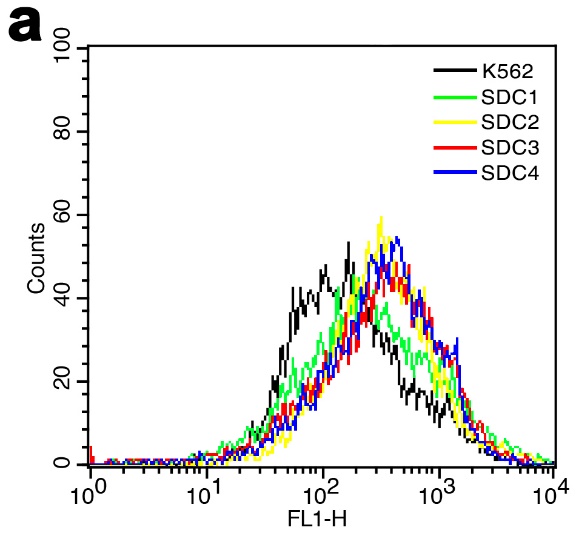


**Supplementary Fig. S2.** Relative HS expression of SDC transfectants. HS expression of stable SDC transfectants created in wild-type (WT) K562 cells was measured by flow cytometry using anti-HS antibody. SDC transfectants with similar amount of HS expression were selected and along with WT K562 cells, applied for further studies. (**a**) Flow cytometry histograms showing HS expression of SDC transfectants and WT K562 cells. (**b**) Detected HS levels were normalized to WT K562 cells as standards. The bars represent mean ± SEM of ten independent experiments. Statistical significance vs WT K562 cells (standards) was assessed by analysis of variance (ANOVA). *p<0.05 vs WT K562 cells; **p<0.01 vs WT K562 cells, ***p<0.001 vs WT K562 cells.


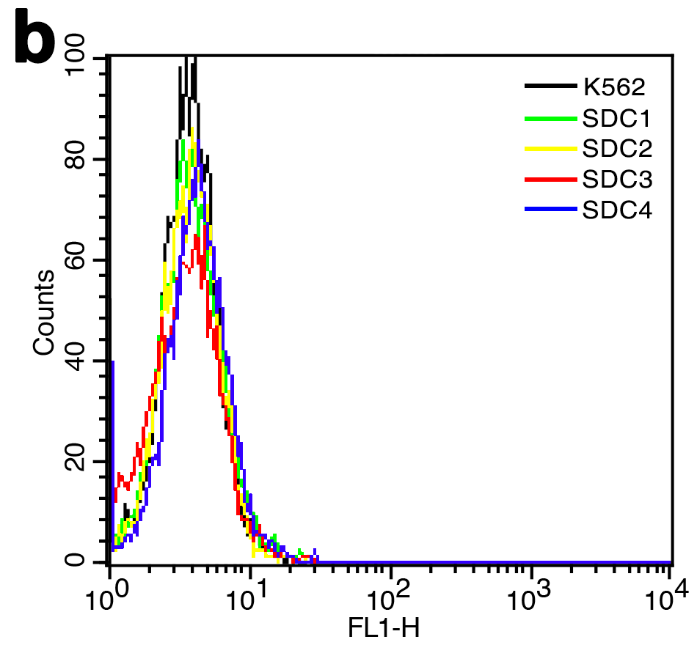

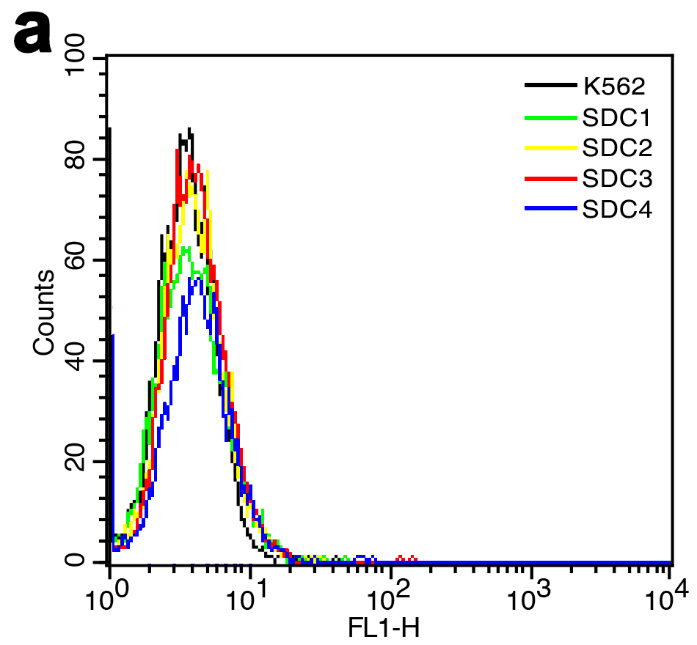


**Supplementary Fig. S3.** Flow cytometric analysis of CS expression in K562 cells and SDC transfectants. CS expression of WT K562 and SDC transfectants was measured by flow cytometry using anti-CS antibody. (**a, b)**: Two representative flow cytometry histograms showing the low CS expression profile of and WT K562 cells SDC transfectants.

**
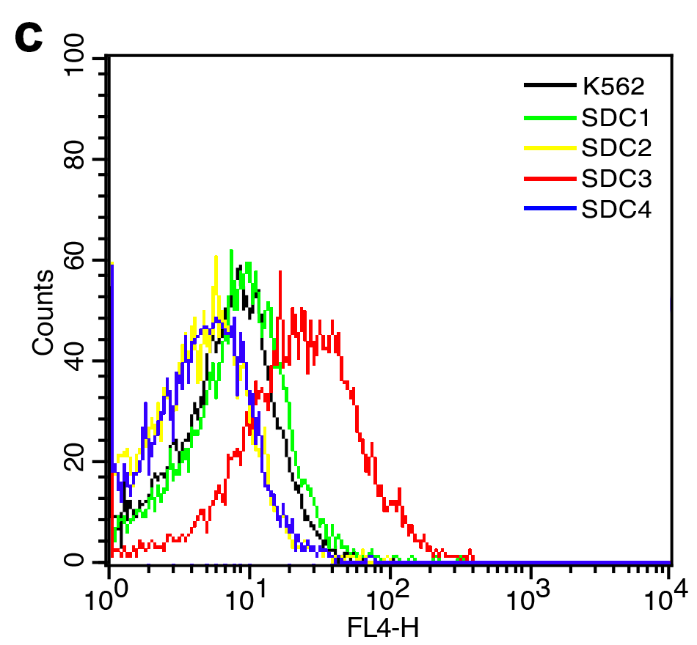

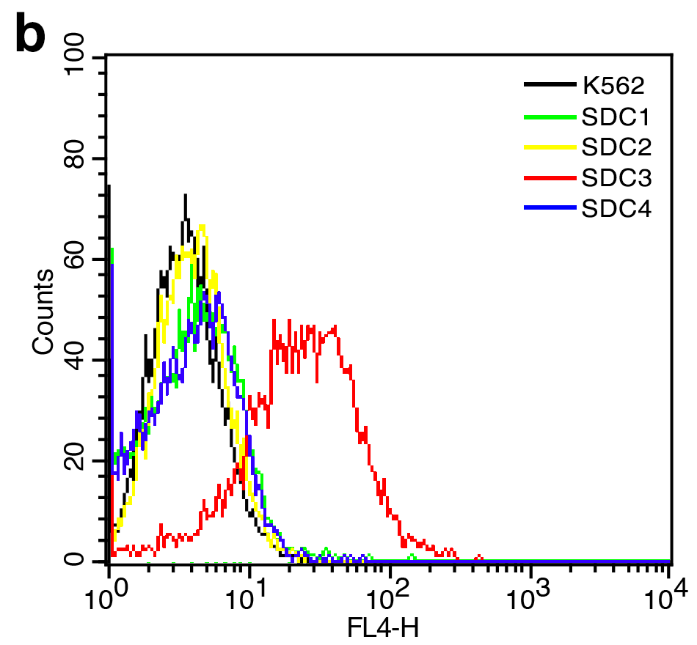

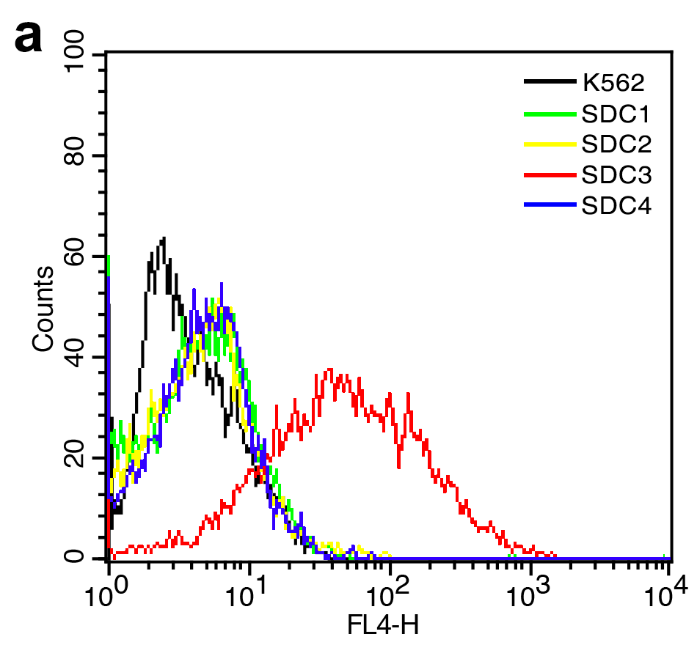
**

**Supplementary Fig. S4.** SDC expression profile of WT SH-SY5Y neuroblastoma cells. SDC expression of WT SH-SY5Y neuroblastoma cells was measured with flow cytometry using APC-labeled anti-SDC antibodies specific for each SDC isoform. (**a-c**) Flow cytometry histograms showing SDC expression of WT SH-SY5Y transfectants. Results of three independent experiments are shown.


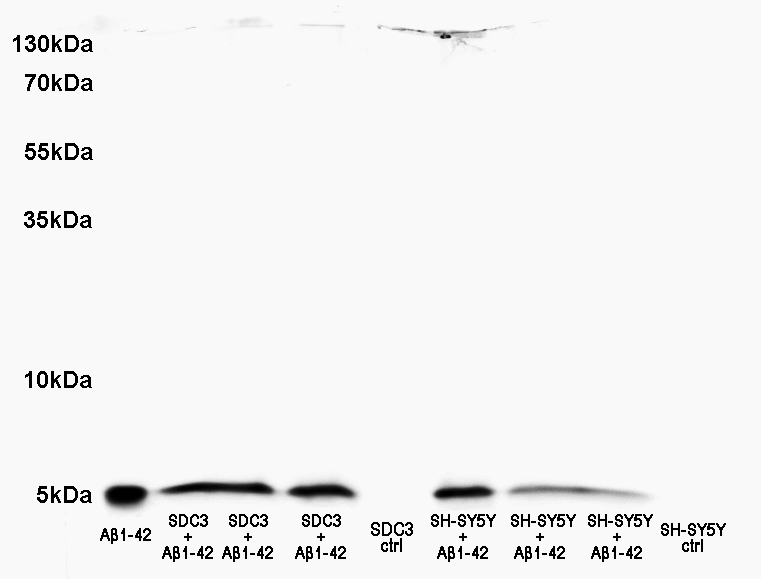


**Supplementary Fig. S5.** SDS-PAGE showing FITC- Aβ1-42 immunoprecipitated with anti-SDC3 antibody from extract of stable SDC3 transfectants (created in K562 cells) or WT SH-SY5Y cells. FITC- Aβ1-42 were detected by Uvitec’s ALLIANCE Q9 ADVANCED imaging platform. Lane 1: 0.5 ug of FITC- Aβ1-42; Lane 2-4: immunoprecipitates of FITC-Aβ1-42-treated stable SDC3 transfectants; Lane 5: immunoprecipitate of untreated SDC3 transfectants (controls); Lane 6-8: immunoprecipitates of FITC- Aβ1-42-treated WT SH-SY5Y cells; Lane 9: immunoprecipitate of untreated WT SH-SY5Y cells (controls). Standard protein size markers are indicated on the left.

| **Supplementary Table S1.** | | | | |  |  |  |  |  |  |  |  |  |  | |  |  |  |
| --- | --- | --- | --- | --- | --- | --- | --- | --- | --- | --- | --- | --- | --- | --- | --- | --- | --- | --- |
| Number of Proteins (After Homology Filtering): **1** | | | | |  |  |  |  |  |  |  |  |  |  | |  |  |  |
| Number of Peptides: **44** | | |  |  |  |  |  |  |  |  |  |  |  |  | |  |  |  |
| Search 1 Number of Data Spectra: **26327** | | | |  |  |  |  |  |  |  |  |  |  |  | |  |  |  |
| Search 1 End Time: **2018-11-15 16:09:27** | | | |  |  |  |  |  |  |  |  |  |  |  | |  |  |  |
| Search 1 Search Time: **14 min 10 sec** | | | |  |  |  |  |  |  |  |  |  |  |  | |  |  |  |
| Search 1 Proteins (Pre Homology Filtering): **8382** Peptides: **38404** | | | | | |  |  |  |  |  |  |  |  |  | |  |  |  |
| Database: **UniProtKB.2015.4.16.random.concat** (77277/47262724 entries searched) | | | | | | | |  |  |  |  |  |  |  | |  |  |  |
| [+] **Original Search Parameters 1** | | | |  |  |  |  |  |  |  |  |  |  |  | |  |  |  |
|  |  |  |  |  |  |  |  |  |  |  |  |  |  |  | |  |  |  |
| Average Parent Error: **0.36** | | |  |  |  |  |  |  |  |  |  |  |  |  | |  |  |  |
| 3 * StdDev: **3.28** | |  |  |  |  |  |  |  |  |  |  |  |  |  | |  |  |  |
|  |  |  |  |  |  |  |  |  |  |  |  |  |  |  | |  |  |  |
| m/z z ppm DB Peptide Variable Mods RT Score Expect # in DB 1076.4576 2 -1.8 **MFFTCGPNEAMVVSGFCR** Acetyl@N-term 90.967 57.0 6.2e-12 8 1055.4550 2 0.63 **MFFTCGPNEAMVVSGFCR** | | | | | | | | | | | | | | |  |  |  |  |
| 73.417 58.2 2.9e-10 8 1115.0316 2 -0.29 **AEAFQMYQEAAQLDMLLEK** | | | | | | |  |  |  |  |  |  |  |  | |  |  |  |
| 84.617 55.4 4.0e-10 4 992.0281 2 1.1 **VASSDLVNMGISVVSYTLK** | | | | | |  |  |  |  |  |  |  |  |  | |  |  |  |
| 88.333 53.5 4.4e-10 6 877.4705 2 0.40 **LPQVAEEISGPLTSANK** | | | | | |  |  |  |  |  |  |  |  |  | |  |  |  |
| 60.167 46.0 1.4e-9 4 1144.0832 2 -0.27 **LAEAEKAQLIMQAEAEAESVR** | | | | | | |  |  |  |  |  |  |  |  | |  |  |  |
| 60.983 57.7 1.5e-9 4 989.9347 2 0.64 **MFFTCGPNEAMVVSGFCR** Met-loss@1 66.567 46.4 1.7e-9 8 1179.0824 2 2.5 **KAEAFQMYQEAAQLDMLLEK** | | | | | | | | | | | | | |  | |  |  |  |
| 78.500 56.6 3.5e-9 4 828.3870 2 0.39 **DIHDDQDYLHSLGK** | | | | | |  |  |  |  |  |  |  |  |  | |  |  |  |
| 41.267 39.4 4.9e-9 7 1009.5225 2 3.8 **TEAEIAHIALETLEGHQR** | | | | | |  |  |  |  |  |  |  |  |  | |  |  |  |
| 65.350 46.3 8.2e-9 6 823.4151 2 0.93 **AQLIMQAEAEAESVR** | | | | | |  |  |  |  |  |  |  |  |  | |  |  |  |
| 59.783 49.8 1.1e-8 4 735.3519 2 1.1 **EMLAAACQMFLGK** | | | | | |  |  |  |  |  |  |  |  |  | |  |  |  |
| 68.717 47.2 3.2e-8 6 690.3654 2 2.6 **ITLVSSGSGTMGAAK** | | | | | |  |  |  |  |  |  |  |  |  | |  |  |  |
| 41.050 46.0 6.0e-8 4 703.4121 2 0.46 **HGVPISVTGIAQVK** | | | | | |  |  |  |  |  |  |  |  |  | |  |  |  |
| 50.450 46.1 1.3e-7 6 709.8857 2 0.28 **AQADLAYQLQVAK** | | | | | |  |  |  |  |  |  |  |  |  | |  |  |  |
| 50.533 45.0 2.2e-7 4 698.3616 2 0.76 **ITLVSSGSGTMGAAK** Oxidation@11 31.567 39.3 2.4e-7 4 882.0007 2 1.0 **LTGVSISQVNHNKPLR** | | | | | | | | | | | | |  |  | |  |  |  |
| 35.250 34.5 2.5e-7 4 903.9462 2 -1.5 **AIMAHMTVEEIYKDR** | | | | | |  |  |  |  |  |  |  |  |  | |  |  |  |
| 48.933 38.7 3.2e-7 6 733.3556 2 0.94 **VSAQCLSEIEMAK** | | | | | |  |  |  |  |  |  |  |  |  | |  |  |  |
| 48.617 42.0 3.4e-7 5 671.3726 3 0.53 **VTGEVLDILSRLPESVER** | | | | | |  |  |  |  |  |  |  |  |  | |  |  |  |
| 80.150 37.5 4.8e-7 4 713.3578 3 1.5 **IQGQNKEMLAAACQMFLGK** | | | | | |  |  |  |  |  |  |  |  |  | |  |  |  |
| 66.550 36.6 7.0e-7 6 735.3894 2 0.57 **AQQVAVQEQEIAR** | | | | | |  |  |  |  |  |  |  |  |  | |  |  |  |
| 30.300 48.8 1.5e-6 4 768.3901 3 0.48 **LAEAEKAQLIMQAEAEAESVR** Oxidation@11 58.483 32.4 1.6e-6 4 750.9218 2 0.54 **VFVLPCIQQIQR** | | | | | | | | | | | | |  |  | |  |  |  |
| 69.233 41.0 2.1e-6 8 591.2932 2 -0.46 **ATYDIEVNTR** | | | | |  |  |  |  |  |  |  |  |  |  | |  |  |  |
| 36.183 36.0 3.0e-6 5 525.6265 3 0.077 **RAQADLAYQLQVAK** | | | | | |  |  |  |  |  |  |  |  |  | |  |  |  |
| 43.717 33.8 4.9e-6 4 655.3416 2 0.98 **KATYDIEVNTR** | | | | |  |  |  |  |  |  |  |  |  |  | |  |  |  |
| 30.617 32.5 7.9e-6 5 601.3433 2 0.57 **VTGEVLDILSR** | | | | |  |  |  |  |  |  |  |  |  |  | |  |  |  |
| 64.917 39.0 1.2e-5 4 608.3696 2 0.95 **ISLNTLTLNVK** | | | | |  |  |  |  |  |  |  |  |  |  | |  |  |  |
| 63.483 38.6 1.3e-5 7 780.4527 2 -1.4 **ISLNTLTLNVKSEK** | | | | | |  |  |  |  |  |  |  |  |  | |  |  |  |
| 55.367 25.6 1.6e-5 7 867.9312 4 -1.2 **EMLAAACQMFLGKTEAEIAHIALETLEGHQR** | | | | | | | |  |  |  |  |  |  |  | |  |  |  |
| 97.783 36.0 1.6e-5 6 562.2928 2 0.060 **QKFSEQVFK** Gln->pyro-Glu@1 45.067 30.2 3.7e-5 7 546.2781 2 0.92 **GEAEAFAIGAR** | | | | | | | | | | | |  |  |  | |  |  |  |
| 41.533 44.5 4.0e-5 4 689.8487 2 0.37 **MRGEAEAFAIGAR** | | | | | |  |  |  |  |  |  |  |  |  | |  |  |  |
| 39.667 43.5 4.7e-5 4 741.3511 2 -1.6 **VSAQCLSEIEMAK** Oxidation@11 45.133 22.6 6.8e-5 5 905.7033 4 -1.0 **VASSDLVNMGISVVSYTLKDIHDDQDYLHSLGK** | | | | | | | | | | | | | | | |  |  |  |
| 98.167 34.2 1.8e-4 6 442.7295 2 0.52 **FSEQVFK** | | | | |  |  |  |  |  |  |  |  |  |  | |  |  |  |
| 38.267 26.3 2.7e-4 7 1321.9954 3 -0.077 **AEAFQMYQEAAQLDMLLEKLPQVAEEISGPLTSANK** | | | | | | | | |  |  |  |  |  |  | |  |  |  |
| 107.500 24.8 4.0e-4 4 570.8055 2 -0.86 **QKFSEQVFK** | | | | |  |  |  |  |  |  |  |  |  |  | |  |  |  |
| 32.333 22.7 9.4e-4 7 813.4400 2 0.57 **AQQVAVQEQEIARR** | | | | | |  |  |  |  |  |  |  |  |  | |  |  |  |
| 26.817 23.9 0.0021 4 478.7798 2 0.0080 **VQVQVVER** | | | | |  |  |  |  |  |  |  |  |  |  | |  |  |  |
| 28.617 29.3 0.0021 4 623.3371 3 -0.91 **QQIEEQRVQVQVVER** | | | | | |  |  |  |  |  |  |  |  |  | |  |  |  |
| 36.117 17.1 0.0028 4 669.3441 2 0.12 **ATYDIEVNTRR** | | | | |  |  |  |  |  |  |  |  |  |  | |  |  |  |
| 30.400 14.4 0.014 5 607.6384 3 0.77 **VSAQCLSEIEMAKAQR** | | | | | |  |  |  |  |  |  |  |  |  | |  |  |  |
| [42.900 17.4 0.019 5](http://193.224.192.33/prospector/cgi-bin/msform.cgi?form=mstag&msms_pk_filter=Max%20MSMS%20Pks&search_key=aGm3dvWmQ48tBZCU&fraction=1&spot_number=42.900&run=1&spectrum_number=1&) | |  |  |  |  |  |  |  |  |  |  |  |  |  | |  |  |  |
|  |  |  |  |  |  |  |  |  |  |  |  |  |  |  | |  |  |  |
| m/z z ppm DB Peptide Variable Mods RT Score Expect # in DB 1313.1627 2 1.0 **SAFSEEVNIKTAEAQLAYELQGAR** | | | | | | | | | | |  |  |  |  | |  |  |  |
| 75.000 49.6 9.1e-11 5 1249.7498 2 -1.4 **LLAELPASVHALTGVDLSKIPLIK** | | | | | | |  |  |  |  |  |  |  |  | |  |  |  |
| 85.650 43.6 2.6e-10 5 1043.0774 2 1.8 **ISAPLTKVDEIVVLSGDNSK** | | | | | | |  |  |  |  |  |  |  |  | |  |  |  |
| 64.933 48.5 3.6e-10 4 967.5518 2 0.34 **LLAELPASVHALTGVDLSK** | | | | | |  |  |  |  |  |  |  |  |  | |  |  |  |
| 71.650 48.5 9.6e-10 5 1088.5441 2 0.31 **CEDVETAEGVALTVTGVAQVK** | | | | | | |  |  |  |  |  |  |  |  | |  |  |  |
| 68.867 56.5 1.1e-9 4 801.4068 2 0.37 **DVYDKVDYLSSLGK** | | | | | |  |  |  |  |  |  |  |  |  | |  |  |  |
| 56.567 45.7 4.2e-9 5 1040.5449 2 0.41 **IMTEKELLAVACEQFLGK** | | | | | |  |  |  |  |  |  |  |  |  | |  |  |  |
| 76.733 44.6 4.7e-9 5 694.8585 2 0.56 **IGEAEAAVIEAMGK** | | | | | |  |  |  |  |  |  |  |  |  | |  |  |  |
| 60.300 51.8 4.6e-8 5 868.4641 2 -0.96 **SILGTLTVEQIYQDR** | | | | | |  |  |  |  |  |  |  |  |  | |  |  |  |
| 70.850 44.1 4.7e-8 5 1017.5182 2 2.0 **TAEAQLAYELQGAREQQK** | | | | | |  |  |  |  |  |  |  |  |  | |  |  |  |
| 45.383 46.2 4.8e-8 5 1163.1092 2 1.2 **SILGTLTVEQIYQDRDQFAK** | | | | | |  |  |  |  |  |  |  |  |  | |  |  |  |
| 70.300 38.5 4.9e-8 5 626.3552 2 1.2 **MGIEILSFTIK** | | | | |  |  |  |  |  |  |  |  |  |  | |  |  |  |
| 83.367 39.0 1.3e-7 5 972.9891 2 0.76 **IGEAEAAVIEAMGKAEAER** | | | | | |  |  |  |  |  |  |  |  |  | |  |  |  |
| 63.900 36.2 1.7e-7 5 689.8944 2 0.94 **NVVLQTLEGHLR** | | | | | |  |  |  |  |  |  |  |  |  | |  |  |  |
| 59.500 45.8 2.2e-7 5 758.9058 2 0.27 **KIGEAEAAVIEAMGK** | | | | | |  |  |  |  |  |  |  |  |  | |  |  |  |
| 54.100 45.8 3.7e-7 5 820.9529 2 1.2 **IRQEEIEIEVVQR** | | | | |  |  |  |  |  |  |  |  |  |  | |  |  |  |
| 45.983 50.3 4.9e-7 5 739.3903 2 0.89 **ELLAVACEQFLGK** | | | | | |  |  |  |  |  |  |  |  |  | |  |  |  |
| 66.900 43.5 6.1e-7 5 760.8897 2 1.2 **TAEAQLAYELQGAR** | | | | | |  |  |  |  |  |  |  |  |  | |  |  |  |
| 51.650 44.8 1.1e-6 5 687.8596 2 0.54 **VDEIVVLSGDNSK** | | | | | |  |  |  |  |  |  |  |  |  | |  |  |  |
| 47.900 38.0 1.6e-6 5 734.4346 2 1.0 **MALVLEALPQIAAK** | | | | | |  |  |  |  |  |  |  |  |  | |  |  |  |
| 79.500 37.4 1.8e-6 5 713.9169 2 0.12 **VKQVLLAQAEAEK** | | | | | |  |  |  |  |  |  |  |  |  | |  |  |  |
| 34.600 44.7 1.8e-6 5 726.4149 2 0.54 **QVLLAQAEAEKIR** Gln->pyro-Glu@1 59.250 40.1 2.2e-6 5 936.4954 2 0.60 **MGIEILSFTIKDVYDK** | | | | | | | | | | | | |  |  | |  |  |  |
| 81.633 29.7 6.2e-6 5 635.3627 2 1.5 **QIAVEAQEILR** | | | | |  |  |  |  |  |  |  |  |  |  | |  |  |  |
| 55.317 30.2 9.0e-6 5 591.8221 2 0.34 **QVLLAQAEAEK** Gln->pyro-Glu@1 55.950 33.9 1.2e-5 5 600.3356 2 0.80 **QVLLAQAEAEK** | | | | | | | | | | | |  |  |  | |  |  |  |
| 37.500 32.9 1.5e-5 5 562.2852 2 0.16 **SAFSEEVNIK** | | | | |  |  |  |  |  |  |  |  |  |  | |  |  |  |
| 41.700 35.7 4.1e-5 5 626.8492 2 1.2 **QIAVEAQEILR** Gln->pyro-Glu@1 72.517 34.6 4.5e-5 5 650.8690 2 1.1 **ISLEIMTLQPR** | | | | | | | | | | |  |  |  |  | |  |  |  |
| 69.367 40.3 4.9e-5 3 686.3599 2 0.82 **QEEIEIEVVQR** | | | | |  |  |  |  |  |  |  |  |  |  | |  |  |  |
| 48.783 32.3 5.0e-5 5 699.4101 2 1.3 **KQIAVEAQEILR** | | | | |  |  |  |  |  |  |  |  |  |  | |  |  |  |
| 46.583 30.1 5.1e-5 5 573.2754 2 0.28 **DADIGVAEAER** | | | | |  |  |  |  |  |  |  |  |  |  | |  |  |  |
| 30.000 38.9 5.2e-5 5 553.2766 3 0.77 **DADIGVAEAERDAGIR** | | | | | |  |  |  |  |  |  |  |  |  | |  |  |  |
| 48.033 27.2 5.8e-5 5 720.7098 3 0.24 **VDEIVVLSGDNSKVTSEVNR** | | | | | | |  |  |  |  |  |  |  |  | |  |  |  |
| 50.183 29.9 9.8e-5 5 491.2665 2 0.62 **VDYLSSLGK** | | | | |  |  |  |  |  |  |  |  |  |  | |  |  |  |
| 49.083 25.1 1.6e-4 5 490.2876 3 0.091 **QVLLAQAEAEKIR** | | | | | |  |  |  |  |  |  |  |  |  | |  |  |  |
| 44.067 23.2 3.5e-4 5 621.8563 2 0.11 **IQQIAEGEKVK** | | | | |  |  |  |  |  |  |  |  |  |  | |  |  |  |
| 21.850 24.5 9.2e-4 5 573.3295 2 -0.59 **TDKELIATVR** | | | | |  |  |  |  |  |  |  |  |  |  | |  |  |  |
| 31.200 30.0 0.0011 5 692.7238 3 0.43 **NVQDIKNVVLQTLEGHLR** | | | | | |  |  |  |  |  |  |  |  |  | |  |  |  |
| 79.833 18.6 0.0018 5 457.2410 2 1.0 **EVAAPDVGR** | | | | |  |  |  |  |  |  |  |  |  |  | |  |  |  |
| 24.633 22.5 0.0057 5 451.7559 2 -0.87 **TQTAVVQR** | | | | |  |  |  |  |  |  |  |  |  |  | |  |  |  |
| 14.717 13.2 0.028 5 500.6068 3 -0.29 **QEEIEIEVVQRK** | | | | |  |  |  |  |  |  |  |  |  |  | |  |  |  |
| [41.983 16.3 0.029 5](http://193.224.192.33/prospector/cgi-bin/msform.cgi?form=mstag&msms_pk_filter=Max%20MSMS%20Pks&search_key=aGm3dvWmQ48tBZCU&fraction=1&spot_number=41.983&run=1&spectrum_number=4&) | |  |  |  |  |  |  |  |  |  |  |  |  |  | |  |  |  |

**Supplementary Table S1.** Sequence of identified peptides characteristic for flotillin- 1 and -2, respectively. Peptide sequences were identified with affinity-based proteomics.
